# TGF-β signaling redirects Sox11 gene regulatory activity to promote partial EMT and collective invasion of oncogenically transformed intestinal organoids

**DOI:** 10.1038/s41389-025-00560-7

**Published:** 2025-05-20

**Authors:** Yu-Hsiang Teng, Bismark Appiah, Geoffroy Andrieux, Monika Schrempp, Katja Rose, Angelika Susanna Hofmann, Manching Ku, Sven Beyes, Melanie Boerries, Andreas Hecht

**Affiliations:** 1 Institute of Molecular Medicine and Cell Research, Faculty of Medicine, University of Freiburg, Freiburg, Germany; 2 Faculty of Biology, University of Freiburg, Freiburg, Germany; 3 Institute of Medical Bioinformatics and Systems Medicine, Medical Center - University of Freiburg, Faculty of Medicine, University of Freiburg, Freiburg, Germany; 4 Department of Pediatrics and Adolescent Medicine, Division of Pediatric Hematology and Oncology, Medical Center - University of Freiburg, Faculty of Medicine, University of Freiburg, Freiburg, Germany; 5 German Cancer Consortium (DKTK), Partner site Freiburg, a partnership between DKFZ and Medical Center - University of Freiburg, Faculty of Medicine, University of Freiburg, Freiburg, Germany; 6 Present Address: Robert Bosch Center for Tumor Diseases (RBCT), Stuttgart, Germany

**Keywords:** Growth factor signalling, Cancer

## Abstract

Cancer cells infiltrating surrounding tissue frequently undergo partial epithelial-mesenchymal transitions (pEMT) and employ a collective mode of invasion. How these phenotypic traits are regulated and interconnected remains underexplored. Here, we used intestinal organoids with colorectal cancer (CRC) driver mutations as model system to investigate the mechanistic basis of TGF-β1-induced pEMT and collective invasion. By scRNA-seq we identified multiple cell subpopulations representing a broad pEMT spectrum, where the most advanced pEMT state correlated with the transcriptional profiles of leader cells in collective invasion and a poor prognosis mesenchymal subtype of human CRC. Bioinformatic analyses pinpointed *Sox11* as a transcription factor gene whose expression peaked in the potential leader/pEMT^high^ cells. Immunofluorescence staining confirmed Sox11 expression in cells at the invasive front of TGF-β1-treated organoids. Loss-of-function and overexpression experiments showed that Sox11 is necessary, albeit not sufficient, for TGF-β1-induced pEMT and collective invasion. In human CRC samples, elevated *SOX11* expression was associated with advanced tumor stages and worse prognosis. Unexpectedly, aside from orchestrating the organoid response to TGF-β1, Sox11 controlled expression of genes related to normal gut function and tumor suppression. Apparently, Sox11 is embedded in several distinct gene regulatory circuits, contributing to intestinal tissue homeostasis, tumor suppression, and TGF-β-mediated cancer cell invasion.

## Introduction

Distant organ metastasis inevitably requires that cancer cells attain the ability to breach normal tissue boundaries and emigrate from the primary tumor. These processes are thought to be facilitated by epithelial-mesenchymal transitions (EMT) in the course of which cancer cells coordinately lose apical-basal polarity and cell-adhesion systems typical of epithelial cells, while gaining front-rear polarity and other mesenchymal features which foster cellular motility and the capacity to invade surrounding stromal tissue [1]. Quite often, though, EMT appears to be only incompletely and reversibly executed, leading to highly dynamic hybrid states or pEMT whereby cancer cells retain to varying degrees epithelial features while additionally procuring mesenchymal characteristics [1, 2]. From a pathophysiological perspective, pEMT is highly disease-relevant, first, because in experimental models of metastasis, cancer cells in pEMT states were found to be much more potent at seeding secondary tumors when compared to cells in fully epithelial or mesenchymal states [3–6]. Second, pEMT has been associated with a collective mode of cancer cell invasion, which is by far the most frequently observed type of stromal infiltration in human cancer [5–10]. Yet, how pEMT and collective invasion are mechanistically interconnected is not clear.

In prototypical instances of EMT, diverse extracellular signals and environmental conditions can trigger expression of members of the Snail, Zeb, and Twist families of transcription factors (TFs) [1]. These core EMT-TFs, in turn, orchestrate widespread gene expression changes that underlie the complementary losses and gains of epithelial and mesenchymal phenotypic traits, respectively. However, a growing number of studies showed that EMT processes may not necessarily unfold as a consequence of a uniform, largely invariant transcriptional program. Instead, large-scale transcriptome analyses of a broad spectrum of human cancers discovered a multiplicity of highly context-dependent and molecularly distinct gene expression programs that predominantly manifest as pEMT states [4, 9, 11–15]. As distinguishing characteristics, these newly described EMT programs often do not show downregulation of epithelial markers in conjunction with upregulation of mesenchymal features that is typically observed in prototypical EMT [11, 13]. Moreover, these new EMT variants apparently can be implemented even when expression of core EMT-TFs is very low or undetectable [4, 9, 11, 13, 14]. In fact, CRISPR/Cas9-mediated knock-out (KO) repeatedly showed that core EMT-TFs are not obligatory for EMT [6, 10, 16–18]. Altogether, evidence is accumulating that aside from prototypical, also mechanistically different, non-prototypical EMT programs exist, which are likely to be driven by TFs other than Snail, Zeb, and Twist family members. This raises the question precisely which TFs operate in different EMT settings.

Sox11 together with Sox4 and Sox12 forms the SoxC subfamily of Sry-related (Sox) TFs [19, 20]. Like all Sox proteins, Sox11 is evolutionary highly conserved and contains a high mobility group (HMG) box domain which facilitates sequence-specific binding to DNA [19, 20]. In addition to its pivotal functions in embryonic development and organogenesis [20], Sox11 emerged as a context-dependent tumor-promoting as well as tumor-suppressing factor with the capacity to both positively and negatively regulate cancer cell proliferation, migration, and invasion [20–26]. In breast cancer, Sox11 was tied to the induction of EMT and acquisition of a hybrid epithelial/mesenchymal state [24, 27]. Similar to other Sox family members with well-established roles in the control of EMT processes [19, 28–31], Sox11, therefore, could be an alternative regulator of EMT processes.

Mouse intestinal organoids carrying colorectal cancer driver mutations in *Apc*, *Kras*, and *Trp53* (TKA-organoids) represent versatile models to investigate various aspects of carcinogenesis. When treated with TGF-β1, TKA-organoids undergo pEMT and form collectively invading cell masses independently of Snail1 and Zeb1 [10]. TKA-organoids thus provide an attractive experimental system highly suitable for the molecular analyses of an intestinal model for pEMT and its relationship to collective invasion. Here, we used single-cell RNA sequencing (scRNA-seq) to comprehensively capture transcriptional and cellular dynamics of TGF-β1-treated TKA-organoids. We found that upon passing through a series of transitory states, cells at the periphery of collectively invading organoids adopted transcriptomic features of leader cells and expressed the highest levels of mesenchymal genes without downregulating epithelial markers. Sox11 emerged as a key driver of TKA-organoid pEMT with additional, TGF-β1-independent gene regulatory activities related to normal gut functions and tumor suppression. In human CRC, expression of mesenchymal markers and *SOX11* turned out to be highly correlated, and elevated *SOX11* expression was paralleled by a worse prognosis. Accordingly, as in TKA-organoids, upregulation of SOX11 might confer mesenchymal and more malignant traits to CRC cells and hence could be a worthwhile drug target.

## Materials and methods

### Organoid culture

Organoids were derived from C57BL/6-*Apc*^*580S/580S*^; *Kras*^*LSL-G12D/+*^; *Trp53*^*LSL-R172H/+*^; *tg(Villin-CreER*^*T2*^*)*/N mice as described [10]. Upon initial establishment, organoids were treated with 0.5 µM 4-hydroxy-tamoxifen (4-OHT; #H7904, Sigma Aldrich, Taufkirchen, Germany) to generate the study-relevant TKA lines (Supplementary Fig. 1). Recombination was verified by PCR (primers listed in Supplementary Table 1).

### Transwell invasion assays

Invasion experiments were conducted as described [10], except that 1 × 10^5^ single organoid cells were dispensed into transwell inserts (#353097, Corning Life Sciences, Corning, New York, USA). After seeding, organoid cells were grown for 30–40 h before treatment with TGF-β1 commenced.

### Genome editing

*Sox11* was inactivated by co-infecting organoids with viral expression vectors for Cas9 and sgRNAs (Supplementary Table 1), which had been designed and cloned into plasmids (listed in Supplementary Table 2) as described [10, 16]. Post infection/selection, single cell-derived organoid clones were screened by PCR (primers listed in Supplementary Table 1). Sox11-HA and Prrx2-HA knock-ins were created following published procedures [32] by co-transfection of sgRNA and CAS9 expression vectors with pCRISPR-HOT-FS-FKBP12^F36V^-HA_2_-P2A-EGFP-loxP-PGK-NeoR-loxP-FS (Supplementary Table 2). After selection of transfected cells with geneticin (10131-027, ThermoFisher Scientific, Waltham, MA, USA), single cell-derived clones were isolated and genotyped by PCR. In-frame insertions of the FKBP12^F36V^-HA_2_-P2A-EGFP-loxP-PGK-NeoR-loxP cassette were confirmed by sequencing.

### Viral transduction

Lentiviral and retroviral particles were produced following published procedures [10] using plasmids listed in Supplementary Table 2.

### Immunofluorescence staining and confocal microscopy

A previously described protocol [10] was employed for whole mount immunofluorescence staining (antibodies listed in Supplementary Table 3) and imaging with a Leica Sp8 confocal microscope.

### RNA isolation, cDNA synthesis, and qRT-PCR

RNA was isolated and further processed for qRT-PCR (primers listed in Supplementary Table 4) following published procedures [10, 16].

### Bulk RNA-seq

Total RNA was sent to Macrogen Inc. (Seoul, South Korea) for library preparation and sequencing. Raw sequencing results were processed on Galaxy EU (Version 0.23.4) (https://usegalaxy.eu/) [33]. Gene expression trajectories were visualized in a heatmap after z-score normalization and k-means clustering [34].

### Pathway analysis

Murine gene names were converted to human identifiers using biomaRt [35]. Pathways and gene sets enriched among differentially expressed genes (DEGs) were identified using Enrichr [36].

### Single-cell RNA-seq

*Preparation of sequencing libraries.* For each experimental condition, 1 × 10^4^ single cells were isolated by flow cytometry and further processed for cDNA library construction using a Chromium chip and a 10x Chromium controller following the Chromium Next GEM Training Kit User Guide (v3.1, CG000204 Rev D; 10x Genomics B.V., Leiden, The Netherlands). Aliquots of the final sequencing libraries were pooled and sent to Macrogen Inc. for sequencing. *Single-cell data preprocessing and alignment.* Cell Ranger was used to align reads and quantify counts based on the reference genome “refdata-gex-mm10-2020-A” (10x Genomics) [37]. Spliced and unspliced reads were quantified with kallisto and bustools programs [38]. The final count matrices served as input for downstream analysis. *Quality control metrics* of count matrices were computed with SCANPY (version 1.9.1) [39]. Cells with fewer than 500 detected genes, more than 40,000 total UMI counts, or greater than 4% mitochondrial counts were discarded. *Normalization, dimensionality reduction, and clustering* Gene expression counts in each cell were log-normalized and scaled by a factor of 10,000 [40]. Principal component analysis (PCA) was performed using the top 5000 highly variable genes. The top 60 principal components were used for neighborhood graph construction. Clustering was performed using the Leiden algorithm [41], with a resolution parameter of 0.5. Uniform manifold approximation and projection (UMAP) [42] was employed for visualization. Highly expressed genes were identified with Wilcoxon rank-sum test. *Differential expression and pathway analysis* DEGs were identified using the *“rank_genes_groups”* function. Genes with an adjusted *p* value < 0.05 and log_2_ fold change ≥ 1 were marked significant. Enrichment analysis was performed using MSigDB Hallmark gene sets and “decoupleR” (version 1.6.0) [43]. *Trajectory inference* Single-cell pseudotime trajectory analysis was done with scvelo (version 0.2.4) [44–46]. Initial and terminal cell states were estimated with CellRank 2 (version 1.5.1) [47]. Partition-based graph abstraction (PAGA) was used to establish connectivities among clusters [48]. *SCENIC* Single-cell regulatory network inference and clustering were performed with pySCENIC (version 0.12.1) [49]. *Consensus Molecular Subtype (CMS) classification* MmCMS (version 0.1.0) [50–52] was used for consensus molecular subtyping of pseudobulk data, which were obtained by pooling scRNA-seq clusters representing the same TGF-β1 treatment times. *Leader cell analysis* For this, average expression of a custom list of 32 leader cell markers (Supplementary Table 5) was scored for each cell with the SCANPY function “tl.score_genes”.

### Protein isolation and western blotting

Cytosolic and nuclear extracts were prepared and used for protein detection by Western blotting with antibodies listed in Supplementary Table 3 as described [10].

### Analysis of TCGA colorectal cancer data

Gene expression data and survival information were retrieved from the UCSC Xena browser (accession date: 23 Jan 2024) [53]. Kaplan–Meier plots were generated using UCSC Xena browser tools. For CMS classification, CMScaller was used [52]. Correlated gene expression and gene expression levels in CMS-stratified samples were computed and plotted by Matplotlib (version 3.8.4) [54] and Seaborn (Version 0.13.2) [55]. Statistical significance was analyzed by Mann–Whitney *U*-tests.

### Statistics and software

Data were processed and visualized using RStudio [56]. Targeted gene expression experiments were analyzed as before [10]. When comparing two populations, statistical significance was assessed using the two-tailed ANOVA test with a confidence interval of 95%. Box plots were generated with ggplot2 [57]. Figures were assembled using Canvas X 2017 (Canvas GFX, Inc.). Schemes depicting experimental designs were generated with BioRender (https://biorender.com/).

Additional information about experimental procedures can be found in the Supplementary Information.

## Results

### TGF-β1 induces a broad spectrum of transition states and a pEMT endpoint in TKA-organoid cells

**Fig. 1:**
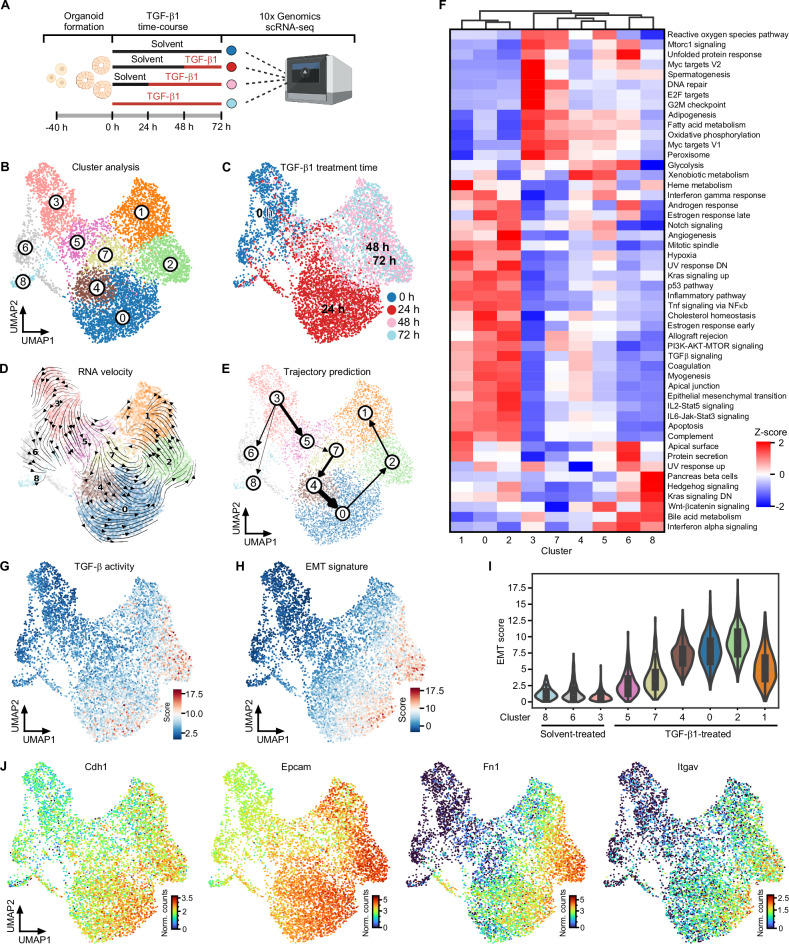
Detection of a broad spectrum of transitory states and a pEMT endpoint in TGF-β1-treated TKA-organoids. **A** Schematic overview of the scRNA-seq workflow. The figure was generated with BioRender.com. **B** Unsupervised clustering and UMAP visualization of 931-TKA organoid cells upon TGF-β1 treatment for 0, 24, 48, and 72 h and scRNA-seq. **C** Individual organoid cells in the UMAP were annotated according to TGF-β1 treatment times deduced by 10x Genomics sample indices. The color code represents TGF-β1 treatment times. RNA velocity analyses (**D**) and PAGA-based trajectory prediction (**E**) of scRNA-seq results from 931-TKA organoids. The arrows in the RNA velocity plot predict the starting point of cell states and directions of future progression projected onto the UMAP space. The thickness of the lines denotes the strength of connectivity between clusters. **F** Heatmap showing the MSigDB hallmark gene sets enriched in gene expression profiles of organoid cell clusters. The red-blue color scale represents the z-score. **G** TGF-β pathway activity was evaluated by MSigDB with decoupleR. Enrichment was determined by over-representation analysis (ORA) using the SCANPY function: score_gene function and visualized in the UMAP. **H** Expression of an EMT signature was scored by MSigDB with decoupleR and projected onto the UMAP space. **I** UMAP cluster-specific visualization of EMT scores in violin plots. **J** UMAPs visualizing expression levels of epithelial and mesenchymal marker genes in organoid cells treated with TGF-β1 for 0, 24, 48, and 72 h. The color code represents the normalized counts (Norm. counts) of the indicated genes.

TGF-β1-treated TKA-organoids form cohesive cell sheets that spread into the surrounding extracellular matrix (Supplementary Fig. 2A) [10]. Concomitantly, they upregulate mesenchymal markers but seemingly maintain epithelial gene expression (Supplementary Fig. 2B, C). Yet, immunofluorescence visualization of TGF-β pathway effectors, a cell polarity marker, and a mesenchymal marker demonstrated some intra-organoid cellular heterogeneity (Supplementary Fig. 2D, E), which might obscure the extent and type of pEMT in bulk analyses. To capture the full range of cellular heterogeneity and gauge the expression of epithelial and mesenchymal markers in individual cells, we performed scRNA-seq with organoids from line 931-TKA [10] which had been exposed to TGF-β1 for different periods of time (Fig. 1A). Following initial processing and quality control of raw sequencing data, a total of 6863 cells allocated to the different treatment groups were available for further examination (Supplementary figure 3). After dimensionality reduction and visualization by UMAP, nine clusters of cells with similar gene expression profiles emerged (Fig. 1B). Comparison with TGF-β1 treatment regimens showed that cells from each treatment group occupied multiple clusters, revealing even greater cellular heterogeneity than anticipated (Fig. 1B, C). Assessment of cell state transitions and trajectory inference using RNA velocity and PAGA identified TGF-β1 treatment-naïve cluster 3 cells as origin of all other cell states (Fig. 1D, E and Supplementary Fig. 4A). Cluster 3 cells are distinguished by enrichment of transcriptomic features associated with cell proliferation and fatty acid metabolism (Fig. 1F), which does not apply to cluster 6 and 8 cells although most of these likewise had not been exposed to TGF-β1. Further, cluster 6 and 8 cells represent developmental lineages distinct from the main trajectory (Fig. 1E, Supplementary Fig. 4A). Along the main trajectory, cells from the 24 h TGF-β1 treatment group populated clusters 5, 7, 4, and 0, while the majority of cells from the 48 h and 72 h TGF-β1 treatment groups were equally partitioned among clusters 2 and 1 (Fig. 1B–E). Clusters 5, 7, 4, and 0 exhibited a gradual decrease in expression of gene sets linked to cell proliferation, while enrichment of gene sets related to TGF-β and other signaling pathways continually increased (Fig. 1F). Enrichment of the TGF-β signaling gene set peaked in cluster 2, which is consistent with UMAP projections of TGF-β pathway activity and TGF-β target gene expression (Fig. 1F, G and Supplementary Fig. 4B). Expression of an EMT-related gene signature followed the same cluster 3→1 trajectory with maximum expression in cluster 2 cells and a subset of cluster 0 cells which might represent precursors of the former (Fig. 1H, I). EMT dynamics of TKA-organoids were paralleled by a gradual transition of their transcriptomes from a profile resembling CMS2 of human CRC to that of CMS4 [58] (Supplementary Fig. 4C). Importantly, though, we could not identify a cell cluster which showed the complementary loss of epithelial and gain of mesenchymal markers commonly observed in prototypical EMT. Rather, UMAP projection of individual gene expression patterns proved co-expression of epithelial and mesenchymal markers at the single cell level, also in cluster 2 cells (Fig. 1J and Supplementary Fig. 4D). In fact, expression of epithelial markers even increased in TGF-β1-treated cells. Altogether, scRNA-seq revealed that TKA-organoid cells, when stimulated with TGF-β1, passed through a continuum of transition states and advanced to a pEMT endpoint reminiscent of non-prototypical pEMT observed in human cancer.

### A leader cell gene expression profile in TKA-organoid cells with the most advanced pEMT state

**Fig. 2:**
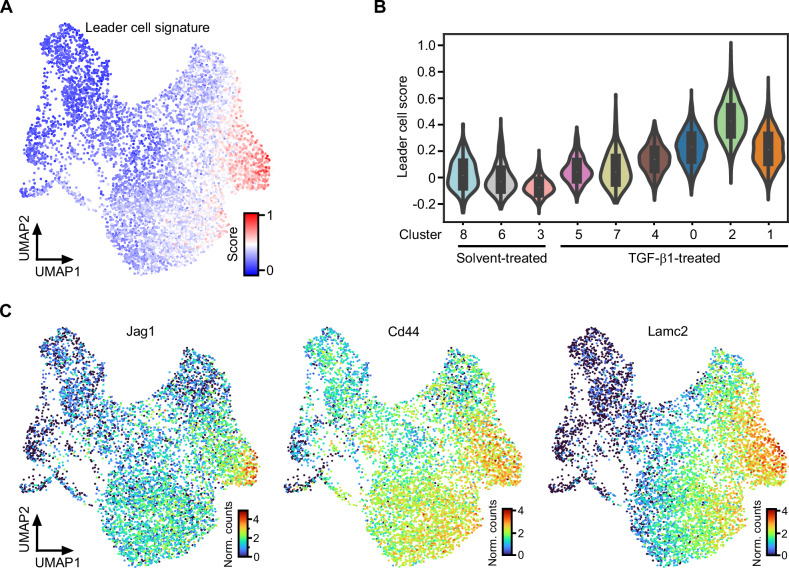
Cells in the most advanced pEMT state express features of leader cells. **A** Expression of a custom leader cell gene expression profile was scored with SCANPY. The obtained leader cell scores were projected onto the UMAP. **B** UMAP cluster-specific visualization of leader cell scores in violin plots. **C** Expression of exemplary leader cell genes visualized in the UMAP space. The color scales represent the normalized counts (Norm. counts) of the indicated genes.

TGF-β1-treated TKA-organoids exhibit a collective mode of invasion in which cells typically segregate into leader and follower populations. Accordingly, scRNA-seq provided an opportunity to examine how the diverse pEMT states adopted by TGF-β1-treated TKA-organoid cells aligned with phenotypically distinct subgroups of collectively invading cell cohorts. To this end, we created a custom gene signature characteristic of leader cells (Supplementary Table 5) and scored its expression in our scRNA-seq dataset. Notably, acquisition of the leader cell gene expression profile occurred progressively and mirrored the dynamics of TGF-β pathway activity and EMT signature expression, with maximum representation in cluster 2 (Fig. 2A, B). This was also evident when transcript levels of individual genes implicated in leader cell formation were examined (Fig. 2C) [4, 59–62]. Moreover, expression of fibronectin, a mesenchymal marker as well as component of the leader cell signature, peaked in the most peripheral cells of TGF-β1-treated TKA organoids, which are likely to represent the leader cell population (Supplementary Fig. 2E). This hints that congruent expression dynamics of the EMT and leader cell signatures are not merely a temporal coincidence and supports the idea that a gradual gain of mesenchymal characteristics is paralleled by leader-follower cell fate decisions whereby the most advanced pEMT state (pEMT^high^) appeared to be associated with leader cells.

### High level *Sox11* expression and regulon activity in pEMT^high^ cells located at the invasive front of TKA-organoids

**Fig. 3:**
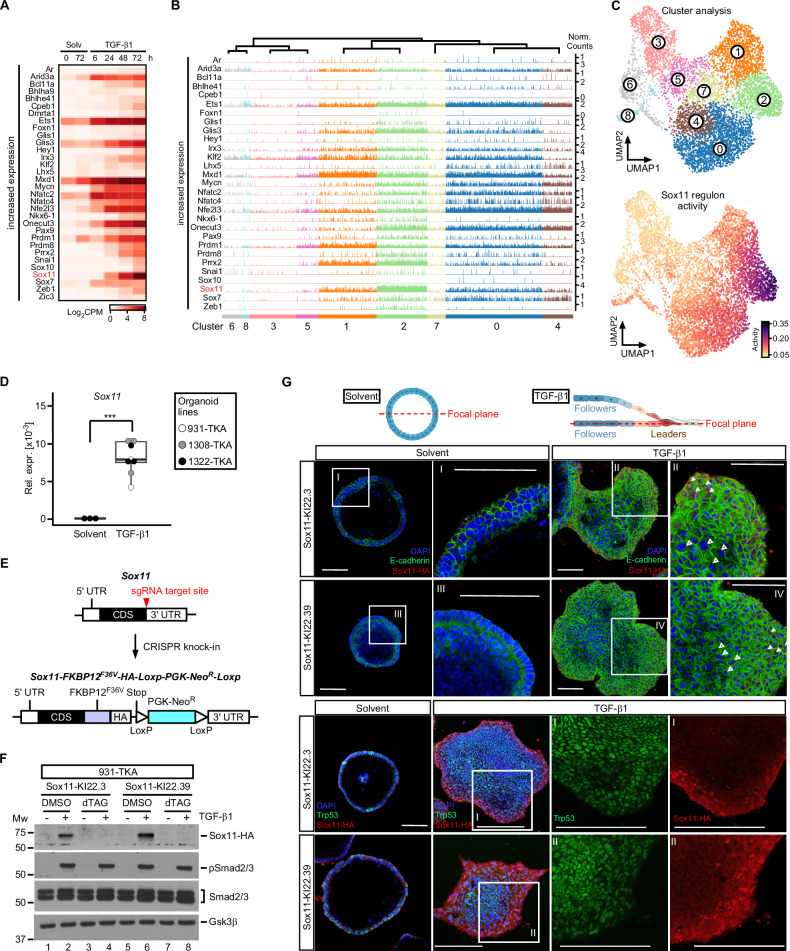
Sox11 is the most highly expressed TGF-β1-inducible transcription factor in cluster 2 cells and localizes to a layer of cells at the invasive front of TKA-organoids. **A** Heatmap displaying TF genes with increasing expression in combined, time-resolved bulk RNA-seq data from solvent (Solv) or TGF-β1-treated 931-TKA and 947-TKA organoids [10]. All murine TFs with log_2_ fold changes > 4 at least at one time point of TGF-β1 stimulation were selected for display. Samples treated with TGF-β1 for 6 h and 24 h were compared to samples harvested at the beginning of the treatment (solvent 0 h). Samples treated with TGF-β1 for 48 h and 72 h were compared to samples kept in solvent for 72 h. Normalized log_2_CPM values were used as gene expression units. **B** Trackplots showing cluster-wise expression of the same TF genes as in (**A**) in scRNA-seq data from solvent or TGF-β1-treated 931-TKA organoid cells. Each peak represents the normalized count for a given gene in a single organoid cell. **C** Unsupervised cell clustering and UMAP visualization of 931-TKA organoid cells upon TGF-β1 treatment for 0, 24, 48, and 72 h (top) and projection of SCENIC-predicted Sox11 regulon activity onto the UMAP (bottom). **D** Expression of *Sox11* in TKA-organoid lines (931-TKA, 1308-TKA, and 1322-TKA). Organoids were embedded in 3 mg/ml Matrigel and treated with solvent or TGF-β1 for 72 h, followed by RNA collection and cDNA synthesis. RNA levels of *Sox11* were evaluated by qRT-PCR and normalized by the expression levels of *Eef1a1*. The box plots show the 26^th^ to 75^th^ percentiles of the data and the median. Dots represent results of individual experiments (*n* = 3). One-Way ANOVA, ***: *p* value < 0.001. **E** Scheme depicting the strategy for CRISPR-mediated in-frame knock-in (KI) of FKBP12^F36V^ coding sequences and the influenza virus hemagglutinin (HA)-epitope tag into the *Sox11* gene. The upper and lower schemes represent WT and modified *Sox11*, respectively. The red arrowhead indicates the sgRNA target site. FKBP12^F36V^ served as a degradation tag. The PGK-promoter driven neomycin resistance gene (Neo^R^) flanked by two loxP sites was used as selection marker. **F** Sox11 expression in Sox11-KI clones. Sox11-KI clones KI22.3 and KI22.39 were treated with solvent or TGF-β1 for 72 h. Two hours before harvest organoids additionally received DMSO or 500 nM dTAG^V^-1. Organoids were collected for western blot analysis to monitor expression of HA-tagged Sox11, phospho-Smad2/3 (pSmad2/3), and total Smad2/3. Gsk3β was detected to control equal loading. Representative results from one out of three experiments are shown (*n* = 3). MW: molecular weight standard in kDa. **G** 931-TKA Sox11-KI organoids were seeded in 3 mg/ml Matrigel and treated with solvent or TGF-β1 for 72 h, followed by whole-mount immunofluorescence staining of the antigens indicated and confocal microscopy. Nuclei were counterstained with DAPI. Positions of focal planes examined are shown in the schemes above the micrographs. Boxed areas with Roman numerals are shown at higher magnification. White arrowheads indicate nuclear Sox11-HA staining at organoid invasive fronts; empty arrowheads indicate absence of Sox11-HA staining in the organoid center. Scale bars: 100 µm. Representative images from one out of three experiments are shown (*n* = 3). The schemes were generated with BioRender.com.

Loss of individual core EMT-TFs does not abrogate TKA-organoid pEMT [10]. To investigate which other TFs might take over their function, we searched for differentially expressed TFs in a previously collected bulk RNA-seq dataset [10], which we expected to provide more complete representation and greater sensitivity than scRNA-seq. Downregulated TF genes included the intestinal stem cell factor *Ascl2*, and the *E2f2* and *Myb* cell cycle regulators (Supplementary Fig. 5A) [63–65]. Diminished expression of these TFs is consistent with the loss of self-renewal capacity and proliferative potential of TGF-β1-treated TKA-organoids (see above; [10]. Among the TF genes upregulated were core-EMT-TFs and several others reported to promote [66–70] or antagonize EMT [71–73] (Fig. 3A). Due to the sparsity of data, expression of some of the TFs could not be followed in scRNA-seq trackplots (Fig. 3B, Supplementary Fig. 5B). Among the TFs for which the trackplots were informative, *Sox11* stood out because *Sox11* transcripts showed the highest fold change in the bulk RNA-seq data, and were particularly abundant in scRNA-seq cluster 2 cells (Fig. 3A, B, and Supplementary Fig. 5C). Yet, since TF expression does not necessarily coincide with function, we additionally assessed TF activity across UMAP clusters by the SCENIC workflow [49]. Supporting validity of this approach, activity of regulons associated with E2f cell cycle regulators was found to be high in cluster 3 (Supplementary Fig. 5D), which had independently been identified as proliferative cell cluster by gene set enrichment analyses (see Fig. 1F). Strikingly, SCENIC assigned the highest activity score in cluster 2 to the Sox11 regulon (Fig. 3C and Supplementary Fig. 5D). Therefore, expression and activity profile marked *Sox11* as a TF gene likely to be critical for the pEMT^high^ state of TGF-β1 stimulated TKA-organoids.

To extend the findings from transcriptome analyses we confirmed that TGF-β1 triggered *Sox11* expression in additional TKA-organoid lines (Fig. 3D). In the absence of commerciably available antibodies suitable for Sox11 protein analyses we inserted a FKBP12^F36V^ cassette with a dual hemagglutinin (HA) epitope tag [74] into the *Sox11* gene (Fig. 3E). This enabled to monitor expression of the resulting Sox11-FKBP12^F36V^-HA (Sox11-HA) fusion by high quality antibodies while dTAG^V^-1-induced degradation of Sox11-HA allowed to control specificity [74, 75]. Indeed, TGF-β pathway activation, demonstrated by Smad2/3 phosphorylation, led to the appearance of an anti-HA-reactive and dTAG^V^-1-sensitive band with the expected molecular weight in western blotting experiments (Fig. 3F). Hence, we proceeded to analyze Sox11-HA spatial distribution by immunofluorescence staining. While solvent-treated controls were uniformly negative, we detected nuclear Sox11-HA in the three to four outermost rows of cells of TGF-β1-treated TKA-organoids (Fig. 3G). The Sox11-HA staining pattern was highly specific, as shown by the disappearance of the signal upon addition of dTAG^V^-1 (Supplementary Fig. 5E) and by comparison with Trp53, which was expressed in all nuclei of TKA-organoids (Fig. 3G). Staining artefacts could be further excluded because a Prrx2-HA fusion protein, also created by knock-in of the FKBP12^F36V^-HA cassette, exhibited an intra-organoid distribution different from Sox11-HA (Supplementary Fig. 5E). Altogether, high-level Sox11-HA expression in a narrow band of nuclei at the organoid periphery confirmed that pEMT^high^ cells with a leader cell gene expression profile reside at the invasive fronts of TGF-β1-treated TKA-organoids.

### Sox11 is required for TGF-β1-induced collective invasion and pEMT

**Fig. 4:**
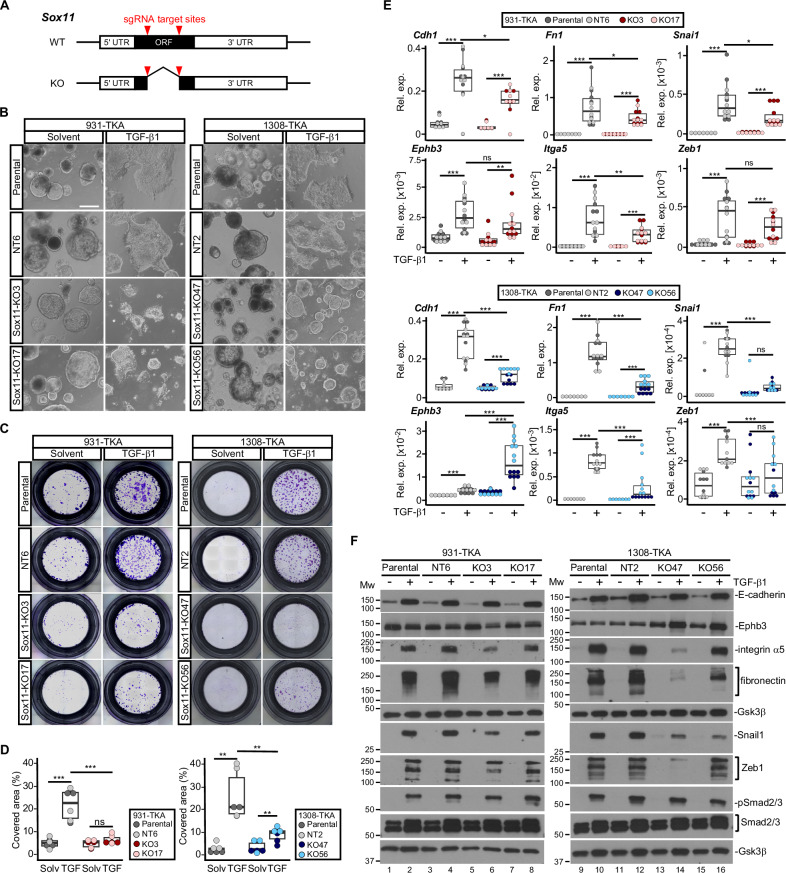
Sox11 is necessary for TGF-β1 induced pEMT and collective invasion. **A** Structures of *Sox11* WT and KO loci. The red arrowheads mark the target sites for two sgRNAs used to delete a large part of the *Sox11* open reading frame (ORF). UTR: untranslated region. **B** Whole-mount phase contrast microscopy of 931-TKA and 1308-TKA organoid lines treated with TGF-β1 for 72 h. The parental lines and clonally derived organoids treated with non-targeting (NT) sgRNAs (931-TKA: NT6; 1308-TKA: NT2) have WT *Sox11* genes. KO lines carry biallellic deletions in *Sox11* (931-TKA: KO3 and KO17; 1308-TKA: KO47 and KO56). Scale bar: 100 µm. Representative pictures from one of three independent biological replicates are shown. **C** Representative images of transwell invasion assays with *Sox11* WT and mutant organoid lines as indicated. Organoids were seeded in 3 mg/ml Matrigel in the upper chambers of transwell inserts and treated with TGF-β1 for 72 h. Invasive organoid cells that had passed through the Matrigel layer and crossed the transwell bottom membrane were visualized by crystal violet staining. **D** Quantification of transwell assays with *Sox11* WT and mutant organoid lines. Areas of transwell membranes covered by invaded organoid cells as exemplarily shown in (**C**) were measured by ImageJ (*n* = 3). Statistical significance was assessed by one-way ANOVA, ns: not significant; **: *p* value < 0.01; ***: *p* value < 0.001. **E** RNA expression levels of epithelial (*Cdh1*, *Ephb3*) and mesenchymal markers (*Itga5*, *Fn1*, *Snai1*, *Zeb1*) in the indicated *Sox11* WT and mutant organoid lines treated with solvent or TGF-β1 for 72 h were determined by qRT-PCR and normalized to those of *Eef1a1* (*n* = 3). The box plots show the 26th to 75th percentiles of the data and the median. Dots represent results of individual measurements. Rel. exp.: relative expression. One-way ANOVA; ns: not significant; *: *p* value < 0.05; **: *p* value < 0.01; ***: *p* value < 0.001. **F** Protein expression levels of epithelial markers (E-cadherin, Ephb3) and mesenchymal markers (integrin α5, fibronectin, Snail1, Zeb1) in the indicated *Sox11* WT and mutant organoid lines treated with solvent or TGF-β1 for 72 h were determined by western blot analyses. Phosphorylated Smad2/3 (pSmad2/3) and total Smad2/3 amounts were analyzed to show TGF-β pathway activation. Detection of Gsk3β served to control equal loading. MW: molecular weight standards in kDa. Images are representative examples from one of three independent biological replicates.

Next, we utilized a doxycycline (Dox-)inducible expression system to test whether overexpression of Sox11 carrying an HA-epitope tag (Sox11^HA^), but not the FKBP12^F36V^ domain, was sufficient to elicit pEMT and collective invasion (Supplementary Fig. 6A). However, Sox11^HA^-expressing TKA-organoids maintained the same cystic shapes as control organoids (Supplementary Fig. 6B, C). Likewise, Sox11^HA^ overexpression neither enhanced organoid invasiveness by itself nor did it alter TGF-β1-induced invasion (Supplementary Fig. 6D–F). Consistent with this, expression of epithelial and mesenchymal markers did not change upon Dox stimulation (Supplementary Fig. 6G). Thus, Sox11^HA^ overexpression did not recapitulate TGF-β1-mediated phenotypic alterations. Nonetheless, Sox11 might still be necessary for pEMT of TKA-organoids. To investigate this, we knocked out *Sox11* in three different TKA-organoid lines by deleting most of the translated region, including the part encoding the HMG-box (Fig. 4A, Supplementary Fig. 7A, B). This deletion abrogated TGF-β1-inducible upregulation of *Sox11* except in one case, although the mutant mRNA of this clone would not be able to give rise to functional protein (Supplementary Fig. 7C). Using for comparison parental lines and *Sox11* wild-type (WT) organoid clones that had been exposed to non-targeting sgRNAs, we examined the TGF-β1 response of the *Sox11* KO organoids. While we observed some line-specific differences in severity of the defects, *Sox11* KO organoids did not flatten and spread as much as control organoids, and in some cases showed signs of disintegration (Fig. 4B and Supplementary Fig. 8A). *Sox11* inactivation also impaired invasive capacities (Fig. 4C, D and Supplementary Fig. 8B, C), and diminished TGF-β1-mediated upregulation of epithelial and mesenchymal markers (Fig. 4E, F and Supplementary Fig. 8D, E). Accordingly, while it did not qualify as an autonomous EMT inducer, Sox11 proved to be a necessary effector of TGF-β signaling to promote pEMT and collective invasion of TKA-organoids.

### Dual impact of *Sox11* on basal and TGF-β1-stimulated gene expression of TKA-organoids

**Fig. 5:**
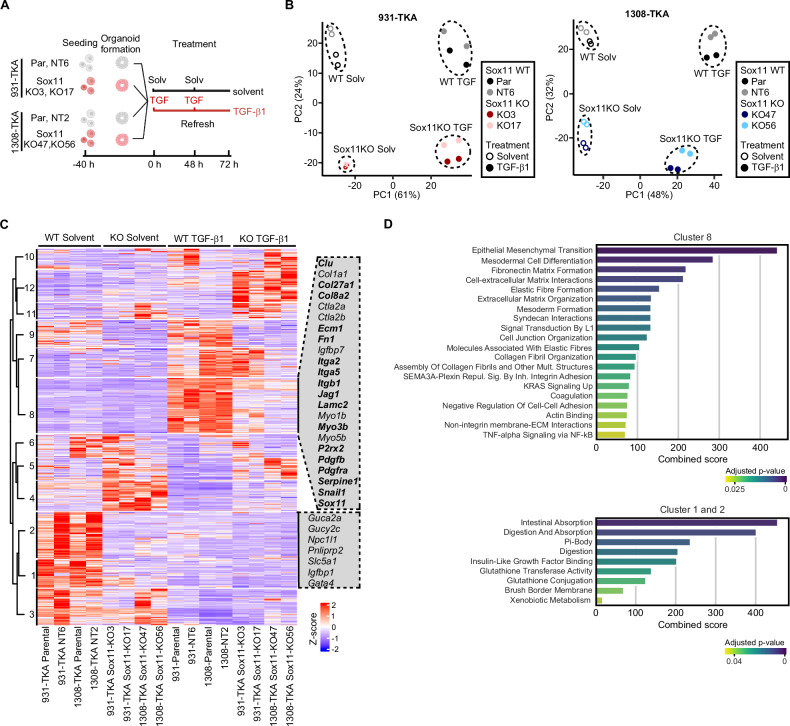
Bulk RNA-seq reveals multiple functions of Sox11 in resting and TGF-β1-stimulated organoid cells. **A** Experimental set-up. *Sox11* WT and mutant organoid lines, as indicated, were seeded in 3 mg/ml Matrigel and treated with solvent or TGF-β1 for 72 h, followed by RNA collection and next generation sequencing. The figure was generated with BioRender.com. **B** Principal component analyses of RNA-seq results from the indicated *Sox11* WT and mutant organoid lines cultured in conditions as shown. The color codes specify *Sox11* genotypes. Empty and filled symbols distinguish samples treated with solvent or TGF-β1 for 72 h. **C** Heatmap showing differentially expressed genes in the indicated *Sox11* WT and mutant organoid lines treated with solvent or TGF-β1 for 72 h. Genes with absolute values of log_2_ fold changes of expression > 1 and adjusted *p* values < 0.05 in any condition when comparing *Sox11* WT and mutant organoid lines were extracted from the bulk RNA-seq results. Gene expression levels in counts per million (CPM) were then normalized by z-score transformation, sorted with a pre-determined number of 12 clusters, and visualized in the heatmap. Selected genes from clusters 1, 2, and 8 are shown next to the heatmap. Bold type identifies members of the *Sox11* regulon with an importance score < 300. The color code indicates z-scores of the genes. **D** Pathway analyses. Differentially expressed genes from clusters 1, 2, and 8 in (**C**) were examined for enrichment of gene expression signatures from the MSigDB Hallmark and Reactomes datasets and the GO collections Biological Process, Cellular Component, and Molecular Function. Results of pathway analyses are given as combined scores based on adjusted *p*-values and odds ratios, which reflect the fraction of genes from a given gene set showing enrichment among DEGs versus the total number of genes in the respective set. Color codes indicate adjusted *p* values with Fisher’s exact test.

To comprehensively characterize the *Sox11*-dependent transcriptome in TKA-organoids, we performed RNA-seq (Fig. 5A). PCA showed that corresponding samples from two independent replicates clustered together, demonstrating experimental reproducibility (Fig. 5B). Separation of samples along PC1 most likely reflects the TGF-β1 response, while separation of *Sox11* WT and KO samples along PC2 hinted at additional, TGF-β1-independent impact of *Sox11* on gene expression. Overall, *Sox11* KO resulted in aberrantly reduced, as well as increased gene expression, arguing that Sox11 acts both as transcriptional activator and repressor in TKA-organoids (Supplementary Fig. 9A). Inter-organoid line comparison additionally demonstrated that TGF-β1-mediated gene expression changes were highly comparable in *Sox11* WT 931-TKA and 1308-TKA organoids whereas transcriptomes of *Sox11*-deficient cells diverged more between the two organoid backgrounds (Supplementary Fig. 9A).

To further examine *Sox11*-regulated transcription, we collected all DEGs in 931-TKA and 1308-TKA organoids and displayed them in a heatmap (Fig. 5C and Supplementary Table 6). This again revealed similarities but also differences (clusters 3, 6, 7, 10) among *Sox11* WT and KO transcriptional profiles of 931-TKA and 1308-TKA organoids (Fig. 5C). Focusing on the TGF-β1 response, clusters 8 and 9 comprised genes with diminished expression in TGF-β1-treated *Sox11*-mutant TKA-organoids. Among these were several genes associated with EMT, TGF-β and Pdgf signaling, actin-myosin filament dynamics, and components of the leader cell signature, some of which are also top-ranking members of the *Sox11* regulon. Pathway analyses likewise indicated that gene sets related to EMT, embryonic development, as well as interaction with and organization of extracellular matrix were the most highly enriched among TGF-β1-regulated genes with diminished expression in *Sox11* KO organoids (Fig. 5D, Supplementary Fig. 10, and Supplementary Table 7). Moreover, bulk RNA-seq cluster 8 contained a sizeable fraction of genes showing comparatively higher expression in scRNA-seq cluster 2 cells (Supplementary Fig. 9B, C). All of this aligns well with the idea of *Sox11* being a key regulator of TGF-β1-induced pEMT and collective invasion of TKA-organoids.

When inspecting *Sox11*-dependent gene expression in TGF-β1-naïve organoids (clusters 1, 2, 4, 5), *Sox11* deficiency was found to diminish expression of several tumor suppressors and genes involved in intestinal function and metabolism, whereas expression of genes related to hypoxia, and interferon signaling was elevated (Fig. 5C, D, Supplementary Fig. 10; Supplementary Table 7) [76–79]. Importantly, *Sox11*-dependent expression of a selection of genes from bulk RNA-seq clusters 1, 2, and 8, and of the tumor suppressor *Gata5*, a paralog of *Gata4* [76], had already been proven by targeted by qRT-PCR analyses (Fig. 4 and Supplementary Fig. 8), or was newly confirmed (Supplementary Fig. 11). In sum, the bulk RNA-seq analyses suggested that *Sox11* might play a hitherto unknown role in intestinal tissue homeostasis and undergoes a TGF-β1-induced switch from potential tumor suppressor to tumor-promoting factor.

### Elevated *SOX11* expression correlates with reduced survival of CRC patients

**Fig. 6:**
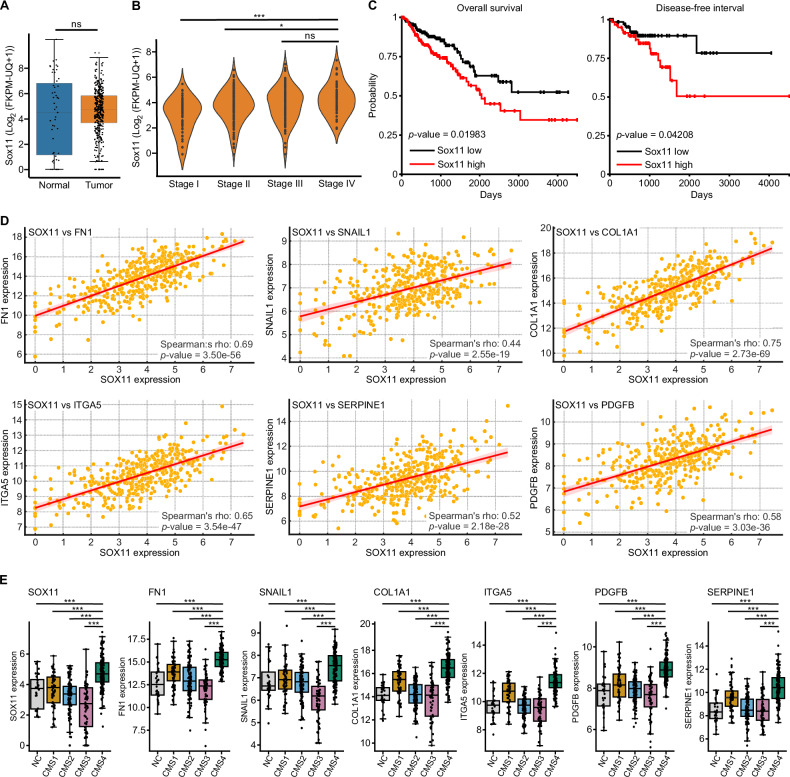
Higher *SOX11* expression correlates with shorter survival times in colon and rectum adenocarcinomas. **A** The gene expression matrices of colon adenocarcinoma (COAD) and rectum adenocarcinoma (READ) samples from the TCGA database were retrieved from the UCSC Xena browser, and *SOX11* RNA expression values in combined COAD and READ cohorts were visualized as box plots. Black dots show *SOX11* expression in individual samples. The box plots show the 26th to 75th percentiles of the data and the median. Statistical analyses were performed using Mann–Whitney *U*-test, ns: not significant. **B** COAD and READ gene expression matrices from the TCGA database were stratified according to tumor stages, and *SOX11* expression levels in combined COAD and READ stage I, II, III, and IV primary tumors are displayed. Black dots show *SOX11* expression in individual samples. Mann–Whitney U test, ns: not significant; *: *p* value < 0.05; **: *p* value < 0.01; ***: *p* value < 0.001. **C** Ka*p*lan-Meier plots showing overall survival and disease-free interval associated with high [log_2_(FPKM-UQ + 1) > 3.842] and low [log_2_(FPKM-UQ + 1) < 3.842] *SOX11* expression in TCGA COAD and READ cohorts. The *p* values were calculated by the log-rank method. **D** Correlation analyses of the expression of the genes indicated in combined transcriptomes of COAD and READ primary tumors. Each orange dot shows an individual sample. A linear regression line (red line), rank-based Spearman correlation coefficients, and *p* values are annotated in each plot. The pink shaded regions indicate 95% confidence intervals. Gene expression levels are given as log_2_(FPKM-UQ + 1) values. **E** Box plots visualizing expression of the genes indicated in CMS-stratified transcriptomes of COAD and READ primary tumors from the TCGA database. Each black dot shows an individual sample. The box plots show the 26th to 75th percentiles of the data and the median. Statistical analyses were performed using Mann–Whitney *U*-test; ***: *p* value < 0.001. NC not classifiable.

To determine whether the findings from TKA-organoids were relevant for human disease, we retrieved gene expression data from healthy tissue and CRC samples from The Cancer Genome Atlas cohorts (TCGA). Although *SOX11* expression in unstratified tumor samples was not significantly higher than in normal tissue, *SOX11* expression steadily increased during tumor progression (Fig. 6A, B). Higher *SOX11* expression was paralleled by reduced overall survival and shorter disease-free intervals (Fig. 6C). Notably, human orthologs of several genes implicated in EMT, TGF-β, and PDGF signaling, which had shown *Sox11*-dependent expression in TKA-organoids, exhibited highly correlated expression with *SOX11* in TCGA samples (Fig. 6D), indicative of some regulatory relationship among these genes also in human cancer. Finally, we examined expression of *SOX11* and the human counterparts of a selection of *Sox11*-dependent genes in colon and rectum adenocarcinoma samples upon CMS stratification [58]. Indeed, especially when compared to CMS2 samples, expression of *SOX11* and several other genes was elevated in CMS4 (Fig. 6E and Supplementary Fig. 12), the subtype with evidence for TGF-β1 pathway activation and mesenchymal characteristics [58], which is consistent with TGF-β1-mediated upregulation of their mouse orthologs in TKA-organoids, and the TGF-β1-induced CMS2 to CMS4 transition of TKA-organoid transcriptional profiles. In summary, the results of these analyses strongly argue in favor of a disease-promoting role for *SOX11* in human CRC.

## Discussion

While cellular plasticity and pEMT provide attractive conceptual frameworks to understand cancer cell metastasis, mechanistic aspects concerning the induction and implementation of pEMT in carcinogenesis remain unclear. This is because gene expression patterns indicative of pEMT in human cancer cells are highly context-dependent and heterogeneous. Often, classical EMT markers are not among DEGs, and core EMT-TFs appear dispensable [11–14]. The preponderance of pEMT over complete EMT further suggests that progression along the epithelial/mesenchymal continuum is limited in vivo [11–14], whereas in vitro EMT models typically advance to fully mesenchymal states [6, 17, 18, 80]. Clearly, novel model systems are needed which accurately reflect recent findings from cancer transcriptomics and allow to generate mechanistic insights required for making the phenomenon of cancer cell pEMT clinically exploitable. Notably, TKA-organoids precisely replicate several aspects of non-prototypical in vivo instances of pEMT, namely persistent expression of epithelial markers, independence at least from single core EMT-TFs, and incomplete execution of EMT. Accordingly, we think that TKA organoids could represent a versatile experimental alternative to complement existing prototypic EMT models.

Despite a broad spectrum of phenotypic manifestations and striking mechanistic differences, there are some features which appear to be shared by the diversity of prototypic and newly emerging variant EMT programs and which are also displayed by the TKA-organoid model. These commonalities include inducibility by TGF-β signaling, gradual progression through a spectrum of transition states towards more mesenchymal phenotypes [3, 5, 6], attenuated proliferation, and changes in metabolic features [16, 81]. A peculiarity of TGF-β1-induced pEMT of TKA-organoids, though, concerns expression of epithelial markers, which is largely maintained and in some cases even increased. While this aligns well with results from gene expression profiling across multiple cancer entities [11–14], it constitutes a noteworthy difference to cell lines models of prototypical EMT and mouse models of pancreatic ductal adenocarcinoma, skin squamous cell carcinoma, and mammary carcinoma where exit from fully epithelial states is accompanied by immediate downregulation for example of *Epcam* and *Cdh1* expression [3, 5].

Currently, it remains unknown why TGF-β1-treated TKA-organoids do not execute a full EMT program and advance to a pEMT state only. Possibly, TGF-β pathway activity does not reach a critical threshold sufficient to enforce completion of EMT due to rapid induction of negative feedback regulators as implied by the temporal profiles of TGF-β-regulated genes. An alternative explanation is suggested by findings that experimental inactivation of core EMT-TFs in pancreatic cancer partly phenocopies TKA-organoid pEMT [6] and that overexpression of Snail1 and Zeb1 cannot trigger EMT in the intestinal organoid model [10]. Hence, functionality of core EMT-TFs may be intrinsically impaired in TKA-organoids. For example, deficiencies in the repressor functions of core EMT-TFs could account for persistent and even elevated expression of epithelial gene expression, including that of epithelial gatekeeper TFs *Foxa1* and *Grhl2* [10, 82, 83], and prevent complete EMT of TGF-β1-treated TKA-organoids. As an ancillary notion, loss of epithelial gene expression seemingly is not a prerequisite for gain of mesenchymal gene expression, questioning whether double-negative feedback loops are inherent regulatory motifs across all EMT variants [80]. Obviously, epithelial and mesenchymal transcriptional programs can co-exist and appear to be driven by mechanistically separable regulatory pathways.

The prevailing type of stromal infiltration in human cancer is collective invasion, whereby invading cell masses segregate into leader and follower cells [7]. The TKA-organoid model corroborated observations from head and neck cancer, pancreatic, lung, and breast cancer that pEMT and collective invasion are interconnected [4, 6, 8, 9]. It also revealed that pEMT^high^ cells are located at the invasive front of TKA-organoids and possess leader cell features. This matches observations in head and neck cancer [4] but differs from a breast cancer model [84], attesting again to context-dependency and variable impact of distinct pEMT states on cancer cell behavior. Notably, TKA-organoid cells gradually acquired leader cell features while advancing towards the pEMT^high^ state without a clear dichotomy between leader and follower cell transcriptomes. However, comparatively minor transcriptional differences between the two cell populations align very well with leader/follower cell plasticity and may facilitate dynamic exchanges of cellular positions and functions in collectively invading cell cohorts [85].

*Sox11* was identified as an essential mediator of TGF-β1-induced pEMT. Beyond this, *Sox11* strongly influenced gene expression in TGF-β1 treatment-naïve TKA-organoids. *Sox11* is transiently expressed in the developing mouse gut [86, 87], but expression and functional importance of *Sox11* in postnatal intestine would be a novel finding extending the list of Sox TFs operating in adult intestinal homeostasis [88]. However, we inactivated *Sox11* in oncogenically transformed cells. Therefore, it needs to be clarified whether the observed transcriptional changes in non-stimulated TKA-organoids reflect a regulatory role of *Sox11* in normal gut rather than a function of *Sox11* acquired during tumorigenesis.

The necessity for *Sox11* for pEMT in a model for intestinal carcinogenesis fits very well with the pro-invasive properties of human *SOX11* in head and neck and breast cancer [23–25, 27]. Interestingly, EMT of murine mammary epithelial cells can also be mediated by *Sox4*, another SoxC family member [30]. SoxC TFs are often co-expressed and may act redundantly [86, 87, 89]. Indeed, *Sox4* and *Sox12* are expressed in TKA-organoids, and both genes respond to TGF-β1 stimulation, albeit in opposite directions (Supplementary Fig. 13). This makes it unlikely that *Sox12* compensates loss of *Sox11*. Still, despite clear-cut effects resulting from *Sox11* inactivation, we cannot rule out that *Sox4* buffers *Sox11* deficiency, explaining the somewhat variable *Sox11* KO phenotype. Nonetheless, our study corroborates the functional importance of SoxC family members in EMT processes, whereby the cellular background may dictate which of them is of greater relative importance.

Sox4 may also serve as paradigm to mechanistically understand the context-dependency of Sox11 gene regulatory functions. Engagement of Sox4 in alternative transcriptional programs involves Sox4 upregulation and reorganization of TF landscapes by TGF-β signaling in pancreatic and mammary cancer cells [28, 31]. Moreover, Sox4 interacts with Smad3, and both TFs are co-bound to *cis*-regulatory elements in TGF-β-treated cells [28, 31]. If applicable to Sox11 and intestinal cells, dosage effects and tight cooperation with TGF-β pathway components would readily explain how Sox11 transcriptional activity might be redirected to promote pEMT. Furthermore, dependency on TGF-β signaling could provide a simple explanation for the inability of Sox11 to autonomously trigger pEMT.

Until now, little is known about *SOX11* in human CRC. Prior studies ascribed both growth-inhibiting and growth-promoting functions to *SOX11* [26, 90]. *SOX11* was also reported to suppress rather than promote CRC cell invasion [21]. Possibly, differences in TGF-β pathway activity and CMS subtype of the experimental models used account for these conflicting results and should be taken into account when testing *SOX11* function. Nonetheless, we believe that the contribution of Sox11 to TGF-β1-induced pEMT and collective invasion in a mouse organoid model for intestinal carcinogenesis reflects cancer-promoting activities of *SOX11* in human colorectal tumors. Future work should aim to substantiate this hypothesis and identify critical targets of SOX11, which may provide new perspectives for CRC treatment.

## Supplementary information

Supplementary Information Teng et al

Teng et al. Supplementary Table 6

Teng et al. Supplementary Table 7

## Data Availability

RNA sequencing datasets were deposited in the Gene Expression Omnibus (accession codes GSE278332 and GSE278490). All other data are presented in the article and its associated Supplementary information, are available from the corresponding author upon reasonable request.

## Code availability

The code used will be made available upon request.
