# Supplementary material for: TGF-β signaling redirects Sox11 gene regulatory activity to promote partial EMT and collective invasion of oncogenically transformed intestinal organoids
Source: Oncogenesis. 2025 May 20;14(1):17. doi: 10.1038/s41389-025-00560-7 (PMC12092639; doi:10.1038/s41389-025-00560-7)
Supplement: Supplementary file 1 — Supplementary Information Teng et al [file 41389_2025_560_MOESM1_ESM.pdf]

Supplementary Information for

TGF-β signaling redirects Sox11 gene regulatory activity to promote partial EMT and collective invasion of oncogenically transformed intestinal organoids

Yu-Hsiang Teng, Bismark Appiah, Geoffroy Andrieux, Monika Schrempp, Katja Rose, Angelika Susanna Hofmann, Manching Ku, Sven Beyes, Melanie Boerries, and Andreas Hecht

This PDF file includes:

- Supplementary methods
- Supplementary tables 1 to 5
- Supplementary figures 1 to 13
- Supplementary references

SUPPLEMENTARY METHODS

Organoid culture. Small intestinal organoid lines were derived from 9-13 weeks old C57BL/6N mice with the genotype *Apc*$^{580S/580S}$; *Kras*$^{LSL\text{-}G12D/+}$; *Trp53*$^{LSL\text{-}R172H/+}$; *tgVillin-CreER*^{T2} as described (1). Organoid line numbers correspond to the identifiers of founder animals (female: #931, #1308, male: #1322). Upon initial establishment, organoids were treated with 0.5 µM 4-hydroxy-tamoxifen (4-OHT; #H7904, Sigma Aldrich, Taufkirchen, Germany) for up to 120 h to concomitantly inactivate *Apc* and remove the lox-stop-lox cassettes from the *Kras*$^{LSL\text{-}G12D/+}$; *Trp53*$^{LSL\text{-}R172H/+}$ loci, thereby generating the TKA lines used in the study (Supplementary figure 1). Recombination was verified by PCR with primers listed in Supplementary table 1 and genomic DNA isolated with the ReliaPrep™ gDNA Tissue Miniprep System (#A2052, Promega, Fitchburg, Wisconsin, USA). For routine growth, TKA-organoids were embedded in a 1:1 mixture of 6 mg/ml Matrigel™ (#356231,Corning Life Sciences, Corning, New York, USA) and 6 mg/ml Cultrex™ BME (#3432-005-01 general or #3533-005-02 type 2; R&D Systems, Bio-Techne GmbH, Wiesbaden, Germany) diluted in Advanced DMEM/F12 (12634-010, Gibco, ThermoFisher Scientific, Waltham, MA, USA) containing 10 mM HEPES (P05-01100, Pan Biotech, Aidenbach, Germany), 100 u each of penicillin/streptomycin (15140-122, Gibco), and 1% (vol/vol) Glutamax (35050-038, Gibco). Matrigel™/Cultrex™ BME plugs were overlaid with culture media containing the ALK2/ALK3 inhibitor LDN193189 (#11802, Cayman Chemical, Ann Arbor, MI, USA) at a final concentration of 50 nM instead of noggin, and no R-spondin-1. Mice were handled in accordance with legal regulations at the Center for Experimental Models and Transgenic Service of the University of Freiburg Medical Center (project registration number: X-21/02F).

Treatment with TGF-β1. For experiments involving TGFβ pathway activation, organoids were seeded in 3 mg/ml Matrigel™. Following a recovery period, organoids received 5 ng/ml human TGF-β1 (#100-21, Peprotech, Rocky Hill, New Jersey, USA) dissolved in PBS supplemented with 0.2% bovine serum albumin. Control cultures received an equivalent volume of the solvent only. Culture media supplemented with growth factors and other reagents were refreshed every 48 h.

Transwell invasion assay. TKA-organoids were disaggregated into single cells by treatment with TrypLE™ Express (12605-010, Gibco) at 37°C for 10 min. Upon neutralization of TrypLE™ Express with PBS and recovery of cells through centrifugation at 290 x g for 3 minutes, single organoid cells were resuspended in 3 mg/ml Matrigel™ and 90 µl aliquots with 1 x 10^5 cells were dispensed into transwell inserts (#353097, Corning Life Sciences). Following matrix solidification at 37°C for 30 minutes and addition of organoid growth medium to the inner and outer transwell chambers, organoids were grown for 30-40 h before treatment with TGF-β1

commenced. At the experimental endpoint, invaded cells on the bottom surface of the membranes were stained with crystal violet followed by image acquisition using a BZ-9000 fluorescence microscope (Keyence, Osaka, Japan), and quantification using ImageJ exactly as described (1).

Genome editing. The single exon gene *Sox11* was inactivated by deletion of a large part of the translated region using two single guide RNAs (sgRNAs; target sites listed in Supplementary table 1) which were designed using the online tool Benchling (https://benchling.com/) and cloned into sgRNA expression vectors listed in Supplementary table 2. TKA-organoids were simultaneously infected with pLenti-Cas9-T2A-BlastR and pLenti-Dest-Sox11-sgRNA2+3-eGFP-F2A-NeoR-loxP. At 48 h post infection, organoids were subjected to combined selection with 7 µg/ml blasticidin (#R210-01, Thermo Fisher Scientific, Waltham, Massachusetts, USA) and 700 µg/ml geneticin (10131-027, Gibco). Upon completion of the geneticin selection, 0.5 µM 4-OHT was administered for 5 days to induce excision of the loxP-flanked sgRNA-expressing provirus. Finally, blasticidin-resistant organoids were dissociated into single cells with TrypLE^{TM} Express and seeded at low densities to facilitate clonal outgrowth and manual picking of single cell-derived organoids. These were expanded and screened by low-input PCR using a protocol established at the ES cell targeting core laboratory, John Hopkins University School of Medicine, Baltimore, MD, USA (https://www.hopkinsmedicine.org/core/ES_Targeting/Protocol_Pages/PCRpicks.html) with primers listed in Supplementary table 1. The monoallelic Sox11-HA and biallelic Prrx2-HA knock-in alleles were created according to published procedures (2, 3). Briefly, 1 x 10^5 single 931-TKA organoid cells were resuspended in 100 µl nucleofection solution consisting of 82 µl buffer L and 18 µl supplement S (Cell Line Nucleofector kit L, #VCA-1005, Lonza, Basel, Switzerland). Using a Nucleofector 2b device and program D-023 (#AAB-1001, Lonza), the cells were co-transfected with 1 µg pCRISPR-HOT-FS-FKBP12^{F36V}-HA$_2$-P2A-EGFP-loxP-PGK-NeoR-loxP-FS together with 0.5 µg pCAS9-mCherry-Frame +1 (3) and 0.5 µg pMuLE ENTR U6-Sox11-KI-sgRNA-CPaint-L1-R5, or with 0.5 µg pCAS9-mCherry-Frame +2 (3) and 0.5 µg pMuLE ENTR U6-Prrx2-KI-sgRNA1-L1-R5. After a recovery period of 48 h, developing organoids were selected with 200 µg/ml geneticin for approximately 10 days. Single cell-derived clones were isolated and genotyped as described above. In-frame insertions of the FKBP12^{F36V}-HA$_2$-P2A-EGFP-loxP-PGK-NeoR-loxP cassette were confirmed by PCR amplification of the 5' and 3' integration sites and sequencing.

Viral transduction. For production of lentiviral and retroviral particles, HEK293T cells were co-transfected with viral vectors and packing plasmids listed in Supplementary table 2 following previously published procedures (1). Virus-containing medium was processed as before and

used for infection of single organoid cells seeded on a Matrigel bed essentially as described (1). Selection with antibiotics started two days after infection, using 7 µg/ml blasticidin, 2 µg/ml puromycin (#P7255, Sigma Aldrich), and 700 µg/ml geneticin, as appropriate. For Dox-inducible gene expression, organoids were co-infected with pMSCV-rtTA3-PGK-NeoR and pRetroX-tight-Sox11-HA-PuroR or pRetroX-tight-MCS-PuroR (1, 4). Expression of $Sox11^{HA}$ was induced with 1 µg/ml Dox (#D9891, Sigma Aldrich).

Immunofluorescence staining and confocal microscopy. For whole mount immunofluorescence staining, mechanically disrupted organoids were seeded into chambered coverslips (#80826, Ibidi, Gräfelfing, Germany), and allowed to recover for 30-40 h before being treated with solvent or TGF-β1 for an additional 72 h. Thereafter, organoids were fixed *in situ* with 4% paraformaldehyde at 4°C for 45 min and further processed using a previously described immunofluorescence staining protocol (1). Primary and secondary antibodies are listed in Supplementary table 3. Immunofluorescence stainings were imaged with a Leica Sp8 confocal microscope with an HC PL APO 20x/0,75 CS2 objective and laser wavelengths of 405, 488, and 561 nm, unless stated differently.

RNA isolation, cDNA synthesis, and qRT-PCR. RNA was isolated with the peqGOLD MicroSpin Total RNA Kit (#12-6831, VWR International GmbH, Bruchsal, Germany) and cDNA was synthesized as described (1, 5). For qRT-PCR, the PerfeCTa® SYBR® Green SuperMix (#95049, Quantabio, VWR) was employed (primers listed in Supplementary table 4) with amounts of cDNA equivalent to 10 ng and 20 ng RNA when PCRs were conducted in CFX384 and CFX96 Touch Real-Time PCR Detection Systems, respectively, (Bio-Rad Laboratories, Feldkirchen, Germany). Raw qRT-PCR data of genes-of-interest were normalized to *Eef1a1* transcripts using the $2^{-\Delta CT}$ method and are presented as relative expression levels.

Bulk RNA-seq. RNA harvested from *Sox11* WT and mutant organoids that had been treated with solvent or TGF-β1 for 72 h, was sent to Macrogen Inc. (Seoul, South Korea) for library preparation and 2 x 151 bp paired-end sequencing using the Illumina TruSeq Stranded RNA Sample Prep Kit (Illumina Inc., San Diego, CA, USA) and an Illumina NovaSeq6000, respectively. Sequencing depth was at least 50 million reads per sample. Raw sequencing results were processed on Galaxy (Version 0.23.4) (https://usegalaxy.eu/) (6). Processing steps included removal of adapter sequences and quality filtering via FastP (Version 0.23.4) (7), followed by read mapping to the mouse reference genome (mmc10) using STAR aligner (Version 2.7.10b). Read counts were normalized to the sequencing depths yielding the counts per millions (CPM) as measures for gene expression levels. DEGs were identified with DEseq2, with adjusted p-values < 0.05 and absolute values of $\log_2$ fold changes > 1 as

thresholds. The regularized log_2 transformation (8) of all gene expression values was used to depict different conditions using principal component analysis (PCA). To visualize gene expression trajectories, the z-score of the CPM values across all samples was calculated and visualized in a heatmap. For k-means clustering, the R package ‘ComplexHeatmap’ was used with the number of clusters set to 12 (9).

Pathway analysis. For pathway analyses of bulk RNA-seq results, murine gene names were first converted to human identifiers using the R/Bioconductor package biomaRt (10). Subsequently, pathways and gene sets enriched among differentially expressed genes were identified using the R/Bioconductor package Enrichr (11). The following collections of functional terms were used for the analysis: GO_Biological_Process_2023, GO_Cellular_Component_2023, GO_Molecular_Function_2023, MsigDB_Hallmark_2020, and Reactome_2022.

Single cell RNA-seq. Preparation of sequencing libraries 931-TKA organoids were cultured in 3 mg/ml Matrigel until they had formed cystic structures. Following this, organoids were treated with solvent or TGF-β1 for up to 72 h. Post-treatment, organoids were dissociated into single cells using TrypLE™ Express. The resultant single organoid cells were washed once with PBS and resuspended in PBS/0.04% bovine serum albumin to enhance cell viability. Larger organoid fragments and cell debris were removed with a 30 µ MACS SmartStrainer (Miltenyi Biotec B.V. & Co. KG, Bergisch Gladbach, Germany). A BD FACSAria™ Fusion flow cytometer was then used to enrich for viable cells of which $1x10^4$ per reaction were prepared for subsequent steps by mixing with the appropriate volumes of RT enzyme, RT reagent, and template switch oligonucleotides according to the Chromium Next GEM Training Kit User Guide (v3.1, CG000204 Rev D; 10x Genomics B.V., Leiden, The Netherlands). These mixtures of cells and reagents were combined with master mix, partitioning oil, and gel beads and loaded into a Chromium chip, which was then sealed with a gasket and processed in the 10x Chromium controller to generate gel beads in emulsion (GEMs). Within the GEMs, samples underwent reverse transcription, followed by a series of clean-up steps, cDNA amplification, and another clean-up step, preparing the cDNA for quality and quantity assessment via an Agilent Bioanalyzer High Sensitivity chip. Adhering strictly to the 10x Genomics user guide, sequencing libraries were prepared which included fragmentation, adaptor ligation, and sample index PCR, with cleanups using SPRIselect beads (Beckman Coulter GmbH, Krefeld, Germany) after each step to ensure the purity and readiness of the libraries for sequencing. Finally, aliquots of the sequencing libraries from solvent and TGF-β1-treated organoid cells were pooled to obtain a final concentration of 10 nM of each of the libraries and the pool was sent to Macrogen inc. for 2 x 150 bp paired-end sequencing on a HiSeq X 150PE, yielding a

total of 800 million paired-end reads (1.6 billion reads in total) for the pooled sample. *Single-cell data preprocessing and alignment* Cell Ranger (10x Genomics, version 6.0.1) (12) was used to construct the reference genome, align, and quantify the counts from single-cell sequencing results adding "--include-introns" flag. Reads were aligned to the mouse reference genome "refdata-gex-mm10-2020-A" (10x Genomics). Spliced and unspliced reads were quantified with kallisto and bustools programs (13) for downstream RNA velocity analysis. Resulting count matrices containing spliced and unspliced read counts were used as input for downstream analysis. *Quality control* Count matrices were loaded and analyzed with SCANPY (version 1.9.1) (14). Quality control metrics were computed, including total number of detected genes per cell, unique molecular identifier (UMI) counts per cell, and percentage of mitochondrial counts. Cells with fewer than 500 detected genes, more than 40,000 total UMI counts, or greater than 4% mitochondrial counts were excluded from further analysis. *Normalization, dimensionality reduction, and clustering* Gene expression counts in each cell were log-normalized and scaled by a factor of 10 000 (15). Principal component analysis (PCA) was performed using the top 5 000 highly variable genes. The top 60 principal components (PCs) were used for neighborhood graph construction. Clustering was performed using the Leiden algorithm (16), with a resolution parameter of 0.5. Uniform manifold approximation and projection (UMAP) (17) was employed for low dimensional visualization of the output from Leiden clustering. Highly expressed genes per cluster were identified with Wilcoxon rank-sum test. *Differential expression and pathway analysis* Differentially expressed genes (DEGs) were identified using *"rank_genes_groups"* function with Wilcoxon rank-sum test. Genes with an adjusted *p*-value < 0.05 and $\log_2$ fold change ≥ 1 were marked significant. Enrichment analysis was performed across clusters using the Hallmark gene set from the mouse MSigDB collections and the Python package "decoupleR" (version 1.6.0) (18). *Trajectory inference analysis* Pseudotime trajectory analysis was performed with scVelo (version 0.2.4) (19–21). In brief, spliced and unspliced read counts were filtered and normalized using default values in the scvelo function "scv.pp.filter_and_normalize". First and second order moments were computed for each cell across 30 nearest neighbors using the first 60 PCs. The final trajectory was visualized in UMAP space. Initial and terminal states of cells were estimated with CellRank 2 (version 1.5.1) (22). Subsequently, partition-based graph abstraction (PAGA) was used to establish the connectivities between clusters in pseudotime space (23). *SCENIC analysis* Single-cell regulatory network inference and clustering was performed as previously described (24) with pySCENIC (version 0.12.1). Transcription factors (TFs) with their associated target genes, collectively termed regulons, were identified based on correlated gene expression across different cells (25, 26). Regulons were then sorted by eliminating target genes that do not show enrichment for corresponding TF binding motif, differentiating direct targets from indirect ones based on the presence of cis-regulatory elements (24, 27).

Consensus Molecular Subtype (CMS) classification Specific clusters from the scRNA-seq generated in this study were pooled to generate pseudobulk gene expression data. The R package MmCMS (version 0.1.0) (28–30) was subsequently used for consensus molecular subtyping of the pseudobulk data. Pooled clusters broadly represent the TGF-β1 treatment time (i.e. clusters 3, 6, 8 – 0 h; clusters 0, 4, 5, 7 – 24 h; clusters 1, 2 – 48 h and 72 h). *Leader cell analysis* To mark and quantify leader cells in the data, a custom list of 32 genes (Supplementary table 5) specifying leader cell identity was used. Average expression of these markers was scored for each cell with the "tl.score_genes" function in SCANPY.

Protein isolation and Western blotting. Organoids were incubated in cell recovery solution (#354253, Corning) and cytosolic and nuclear extracts were prepared by resuspending organoids in nuclear extraction buffer A (10 mM HEPES/KOH pH 7.9, 0.1 mM EDTA, 10 mM KCl, 1x Complete® [1697498, Roche® Life Sciences, Merck KGaA, Darmstadt, Germany], 1 mM DTT, 1x phosphatase inhibitor cocktails 2 and 3 [#P5726/#P0044, Sigma Aldrich]). After incubation on ice for 15 min, 0.5% (v/v) NP-40 was added, organoid suspensions were shortly vortexed, and nuclei were pelleted by centrifugation at 4°C and 16 100 x g for 2 min. The cytosolic supernatant was collected, and the nuclear pellet was washed once with nuclear extraction buffer A. Thereafter, nuclei were resuspended in 20 mM HEPES/KOH pH 7.9, 400 mM NaCl, 1 mM EDTA, 1x Complete®, 1 mM DTT, 1x phosphatase inhibitor cocktails 2 and 3, and incubated at 4°C for 30 min with constant shaking. Nuclear extracts were cleared by centrifugation at 4°C and 16 100 x g for 10 min. Protein concentrations were determined with the BioRad DC™ Protein Assay (#500-0113, BioRad, Feldkirchen, Germany). Depending on the protein yield in a given experiment, 25-40 µg of protein was separated by SDS-PAGE and transferred to nitrocellulose for protein detection as described (31). Antibodies are listed in Supplementary table 3.

Analysis of TCGA colorectal cancer data. Processed gene expression data sets and survival information from the TCGA COAD and READ cohorts were retrieved from the data hub on the UCSC Xena browser (accession date: Jan 23, 2024) (32). Survival analysis and visualization by Kaplan-Meier plots were done using UCSC Xena browser tools. For CMS classification the R/Bioconductor package CMScaller was used (30). Correlated gene expression, and gene expression levels in CMS-stratified samples were computed and plotted by Matplotlib (version 3.8.4) (33) and Seaborn (Version 0.13.2) (34). Statistical significance was analyzed by Mann-Whitney U tests.

Statistics and software. Data were analyzed and visualized using RStudio (35). For targeted gene expression studies, statistical analyses were done using a linear model as before (1).

When comparing two populations, statistical significance was assessed using the two-tailed ANOVA test with a confidence interval of 95%. Box plots were generated with ggplot2 (36) and display the median with the lower and upper quartile. Whiskers show 1.5 times the interquartile range. Final figures were assembled using Canvas X 2017 (Canvas GFX, Inc.). Schemes depicting experimental designs of scRNA-seq, bulk RNA-seq, the Dox-inducible overexpression system, immunofluorescence experiments and transwell assays were generated with BioRender (https://biorender.com/) with publication licenses.

Supplementary table 1:

DNA sequences of sgRNA targets and oligonucleotides employed in genome editing experiments (5’-3’)

**sgRNA target sequences including PAM***	
Sox11-sgRNA2	GAAGATCCCGTTCATCAGGGAGG
Sox11-sgRNA3	GTGTCCACCTCCTCATCCAGCGG
Sox11_KI_sgRNA_CPaint	CCTGGTGTTCACGTATTGAGAGG
Prrx2_KI_sgRNA1	AATGCACAGTCAGTTCACTGTGG
non-targeting-sgRNA-L1	CATACCCGCGCCGTGACTCC
non-targeting-sgRNA-R1	GGACGGATGGGACGACTAGT
**PCR primers for genotyping**	
Apc-A1	GTTCTGTATCATGGAAAGATAGGTGGTC
Apc-A2	GAGTACGGGGTCTCTGTCTCAGTGAA
Apc-A3	CACTCAAAACGCTTTTGAGGGTTG
Kras-K1	GTCTTTCCCCAGCACAGTGC
Kras-K2	CTCTTGCCTACGCCACCAGCTC
Kras-K3	AGCTAGCCACCATGGCTTGAGTAAGTCTGCA
Trp53-T1	AGCCTTAGACATAACACACGAACT
Trp53-T2	CTTGGAGACATAGCCACACTG
Trp53-T3	GCCACCATGGCTTGAGTAA
Sox11_sPCR_Fwd1	GATCGAGCGCAGGAAGATCA
Sox11_sPCR_Rev1	GTGCAGTAGTCGGGGAACTC
Sox11-int-del-R	CGTCATCTTCGTCGTCGTCT
Sox11-F3	AGGACCTGGATTCCTTCAGC
Sox11_KI_screen_for	CTGGAGGCGAACTTCTCCGA
Prrx2_intron3_P11	CTGGGACCTGTGTTTCCTGT
Prrx2_exon4_P12	CTCAGCCACCATAGCAGTGA
Prrx2_KI_screen_for	CCTCAAGGCCAAAGAGTTCA
FKBP1-R1	ATGTGGTGGGATGATGCCTG
Neo_KI_screen_for	GCGAATGGGCTGACCGCTTC
Sox11-R3	AATTTCTCAGCGCCACATCT
EGFP_seq_rev	TCCAGCTCGACCAGGATGGGC

*: PAM is shown where applicable

Supplementary table 2:

Plasmids used in the study

**Plasmid** ***(purpose)***	**derived from**[a]	**Source(s) of parental vectors**
psPAX2 *(lentiviral packaging)*	n.a.	a gift from Didier Trono: Addgene plasmid #12260; RRID:Addgene_12260
pMD2.G *(lentiviral packaging)*	n.a.	a gift from Didier Trono: Addgene plasmid #12259; RRID:Addgene_12259
Hit60 *(retroviral packaging)*	n.a.	a gift from Ulrich Maurer, University of Freiburg (37)
pVSV-G *(retroviral packaging)*	n.a.	(ClonTech, 631530)
pMSCV-RIEP *(stable expression of rtTA3)*	n.a.	a gift from Cornelius Miething, University of Freiburg (38)
pMSCV-rtTA3-PGK-NeoR *(stable expression of rtTA3)*	pMSCV-RIEP	a gift from Marion Flum, University of Freiburg
pRetroX-tight-MCS-PuroR	n.a.	(4)
pRetroX-tight-Sox11-HA-PuroR *(Dox-inducible expression of Sox11-HA)*	pRetroX-Tight-mLEF1m5Mut-HA-Puro	(39)
pLenti-Cas9-T2A-BlastR *(viral Cas9 expression vector)*	n.a.	a gift from Jason Moffat; Addgene plasmid #73310; RRID:Addgene_73310
pMuLE ENTR U6-Sox11-sgRNA2-L1-R5 *(sgRNA expression vector)*	pMuLE ENTR U6 stuffer sgRNA scaffold L1-R5	a gift from Ian Frew; Addgene plasmids # 62127; RRID:Addgene_62127
pMuLE ENTR U6-Sox11-KI-sgRNA-CPaint-L1-R5 *(sgRNA expression vector)*		
pMuLE ENTR U6-Prrx2-KI-sgRNA1-CPaint-L1-R5 *(sgRNA expression vector)*		
pMuLE ENTR U6-Sox11-sgRNA3-L5-L2 *(sgRNA expression vector)*	pMuLE ENTR U6 stuffer sgRNA scaffold L5-L2	a gift from Ian Frew; Addgene plasmids #62130; RRID:Addgene_62130
pLenti-Dest-Sox11-sgRNA2+3-eGFP-F2A-NeoR-loxP *(viral expression vector for Sox11-targeting sgRNAs)*	pMuLE-Lenti-Dest-eGFP-NeoR-LoxP	a gift from Ian Frew; Addgene plasmid #62175; RRID:Addgene_62175
pCAS9-mCherry-Frame +1 *(viral Cas9 expression vector)*	n.a.	a gift from Veit Hornung; Addgene plasmid # 66940; http://n2t.net/addgene:66940; RRID:Addgene_66940)
pCAS9-mCherry-Frame +2 *(viral Cas9 expression vector)*	n.a.	a gift from Veit Hornung; Addgene plasmid # 66941; http://n2t.net/addgene:66941; RRID:Addgene_66941)
pCRISPR-HOT-FS-FKBP12^{F36V}-HA$_2$-P2A-EGFP-loxP-PGK-NeoR-loxP-FS	pCRISPR-HOT_tdTomato	a gift from Hans Clevers; Addgene plasmid # 138567;

(vector for knock-in of FKBP12^{F36V}-HA$_2$ cassette into Sox11 gene)		http://n2t.net/addgene:138567; RRID:Addgene_138567

[a]: all plasmids were generated by standard cloning techniques; details are available upon request from the corresponding author.

n.a.: not applicable

Supplementary table 3:

List of antibodies used for immunofluorescence staining and Western blotting

**Primary antibodies for immunofluorescence staining**		
**Antigen**	**Type, origin, dilution**	**Catalogue number, clone number*, supplier**
β-Catenin	polyclonal, rabbit, 1:50	#9581, Cell Signaling Technology, Danvers, Massachusetts, USA
E-cadherin	monoclonal, mouse, 1:200	#610182, clone 36, BD Biosciences, San Jose, California, USA
Fibronectin	polyclonal, rabbit, 1:400	#ab2413, Abcam, Cambridge, UK
PKC ζ (atypical PKC isoform)	monoclonal, mouse, 1:100	#sc-17781, clone H-1, Santa Cruz Biotechnology, Heidelberg, Germany
p53	monoclonal, mouse, 1:200	#2524, Cell Signaling Technology, Danvers, Massachusetts, USA
Smad2/3	monoclonal, rabbit, 1:800	#8685, clone D7G7, Cell Signaling Technology, Cambrige, UK
HA-tag	polyclonal, rabbit, 1:250	#ab9110, Abcam, Cambridge, UK
**Secondary antibodies for immunofluorescence staining**		
**Antigen**	**Origin, fluorophore, dilution**	**Catalogue number, supplier**
mouse IgG	donkey, Alexa Fluor488-conjugated, 1:200	#A-11001 Invitrogen, Carlsbad, California, USA
mouse IgG	donkey, Alexa Fluor555-conjugated, 1:500	#A-31570, Thermo Fisher Scientific, Dreieich, Germany
rabbit IgG	goat, Alexa Fluor555-conjugated, 1:500	#A-11064 Invitrogen, Carlsbad, California, USA
rabbit IgG	goat, Alexa Fluor488-conjugated, 1:200	#A-11008, Thermo Fisher Scientific, Dreieich, Germany
**Primary antibodies for Western blotting**		
**Antigen**	**Type, origin, dilution**	**Catalogue number, supplier, clone number***
E-cadherin	monoclonal, mouse, 1:1000	#610404, BD Biosciences, San Jose, California, USA
Ephb3	monoclonal, mouse, 1:5000	#H00002049-M01, clone 3F12, Abnova, Taipei, Taiwan
Fibronectin	polyclonal, rabbit, 1:1000	#ab2413, Abcam, Cambridge, UK
Gsk3β	monoclonal, mouse, 1:1000	#610201, clone 7, BD Biosciences, San Jose, California, USA
anti-HA	monoclonal, rat, 1:1000	#11867423001, 3F10, Roche® Life Sciences, Merck KGaA, Darmstadt, Germany
Integrin-α5	polyclonal, rabbit, 1:1000	#4705, Cell Signaling Technology, Cambrige, UK
Phospho-Smad2 (Ser465/467)/Smad3 (Ser423/425)	monoclonal, rabbit, 1:1000	#8828, clone D27F4, Cell Signaling Technology, Cambrige, UK
Smad2/3	monoclonal, rabbit, 1:5000	#8685, clone D7G7, Cell Signaling Technology, Cambrige, UK
Snail1	monoclonal, rabbit, 1:1000	#3879, clone C15D3, Cell Signaling Technology, Cambrige, UK
Zeb1	monoclonal, rabbit, 1:1000	#70512, XP®, clone E2G6Y, Cell Signaling Technology, Cambrige, UK
**Secondary antibodies for Western blotting**		

Antigen	Type, origin, dilution	Catalogue number, supplier, clone number*
mouse IgG	polyclonal, goat, HRP-conjugated, 1:10000	#115-035-146, Dianova, Hamburg, Germany
rat IgG	polyclonal, goat, HRP-conjugated, 1:10000	#112-035-062, Dianova, Hamburg, Germany
rabbit IgG	polyclonal, goat, HRP-conjugated, 1:10000	#111-035-045, Dianova, Hamburg, Germany

*: if applicable

Supplementary table 4:

List of primer sequences used for qRT-PCR

Gene	forward (5´ - 3´)	reverse (5´ - 3´)
Cdh1	CTTTAAGCCCAGCACTCAGG	CCTGCTTCCTGAGAAAATGC
Clu	AGCCGTGCGGAATGAGATAG	GTTCTCTAGGGGTCTGCAGC
Ctla2a	GCTCAGCCAGAGTAACAGCT	CTCCTGCTGTTGAGCCTCTC
Eef1a1	GACAGCAAAAACGACCCACC	GGGCCATCTTCCAGCTTCTT
Ephb3	GGTTTGCATCCTTTGACCTG	CTCGTTGGAGCTGAGTGTCA
Fn1	TGACGCTGGCTTTAAGCTCA	TCATCCGCTGGCCATTTTCT
Gata4	CTGTGCCAACTGCCAGACTA	TTTGAATCCCCTCCTTCCGC
Gata5	AAGCAGGCATACCTCACCAC	CCAGGTCTCTAGTCCCCTCC
Guca2a	GCCACTCTGCACTTCCAGAA	CTAGCATCCCGTACAGGCAG
Itga5	ATTTCCGAGTCTGGGCCAAG	GATCCACAACGGGACACCAT
P2rx2	CCACCACCACTCGAACTCTC	GTCAGAGCAGTGGCCAGATT
Pdgfb	CGGCCTGTGACTAGAAGTCC	CCTTGTCATGGGTGTGCTTA
Pnliprp2	GTGGGTACATCTTGGTCGCT	GCTTCTGGTTGGAGGGATCC
Serpine1	AGCCTTTGTCATCTCAGCCC	GCGTCTCTTCCCACTGTCAA
Slc5a1	TCACCAAGCCCATTCCAGAC	GGAGTCCTCTGGGATGTCCT
Snai1	CTTGTGTCTGCACGACCTGT	CTTCACATCCGAGTGGGTTT
Sox4	GGGCAGTTTCAGCTCCTCAT	CCAGCCAATCTCCCGAGATC
Sox11	GAGTTCCCCGACTACTGCAC	CCATGAGCATCTTCTCCCCC
Sox12	CTTCGGGCACGTCACATTTC	TAGGTGAAAACCAGGTCGGC
Zeb1	GGGGCATCTCACACTTTTGT	AACGGCTGTGAACCAAAAAC

Supplementary table 5:

Components of the leader cell gene expression signature

Gene name	References
Ddr2<br>Cxcr4	Hwang, P.Y., Brenot, A., King, A.C., Longmore, G.D., and George, S.C. (2019). Randomly Distributed K14+ Breast Tumor Cells Polarize to the Leading Edge and Guide Collective Migration in Response to Chemical and Mechanical Environmental Cues. Cancer Res 79, 1899-1912.
Snai1<br>Vim<br>Twist1<br>Zeb1	Quan, Q., Wang, X., Lu, C., Ma, W., Wang, Y., Xia, G., Wang, C., and Yang, G. (2020). Cancer stem-like cells with hybrid epithelial/mesenchymal phenotype leading the collective invasion. Cancer Sci 111, 467-476.
Krt14	Cheung, K.J., Gabrielson, E., Werb, Z., and Ewald, A.J. (2013). Collective invasion in breast cancer requires a conserved basal epithelial program. Cell *155*, 1639-1651.
Vim<br>Pdpn<br>Lamc2<br>Lamc3<br>Mmp10<br>Tgfbi<br>Itga5	Puram, S.V., Tirosh, I., Parikh, A.S., Patel, A.P., Yizhak, K., Gillespie, S., Rodman, C., Luo, C.L., Mroz, E.A., and Emerick, K.S., et al. (2017). Single-Cell Transcriptomic Analysis of Primary and Metastatic Tumor Ecosystems in Head and Neck Cancer. Cell *171*, 1611-1624.e24.
Cdh2	Saénz-de-Santa-María, I., Celada, L., and Chiara, M.-D. (2020). The Leader Position of Mesenchymal Cells Expressing N-Cadherin in the Collective Migration of Epithelial Cancer. Cells 9, 731.
Ctsb	Wu, J.-S., Li, Z.-F., Wang, H.-F., Yu, X.-H., Pang, X., Wu, J.-B., Wang, S.-S., Zhang, M., Yang, X., and Cao, M.-X., et al. (2019). Cathepsin B defines leader cells during the collective invasion of salivary adenoid cystic carcinoma. Int J Oncol 54, 1233-1244.
Cx43	Khalil, A.A., Ilina, O., Vasaturo, A., Venhuizen, J.-H., Vullings, M., Venhuizen, V., Bilos, A., Figdor, C.G., Span, P.N., and Friedl, P. (2020). Collective invasion induced by an autocrine purinergic loop through connexin-43 hemichannels. J Cell Biol *219*.
Cldn11	Li, C.-F., Chen, J.-Y., Ho, Y.-H., Hsu, W.-H., Wu, L.-C., Lan, H.-Y., Hsu, D.S.-S., Tai, S.-K., Chang, Y.-C., and Yang, M.-H. (2019). Snail-induced claudin-11 prompts collective migration for tumour progression. Nat Cell Biol *21*, 251-262.
Vegfa<br>Dll4<br>Cdh5	Konen, J., Summerbell, E., Dwivedi, B., Galior, K., Hou, Y., Rusnak, L., Chen, A., Saltz, J., Zhou, W., and Boise, L.H., et al. (2017). Image-guided genomics of phenotypically heterogeneous populations reveals vascular signalling during symbiotic collective cancer invasion. Nat Commun *8*, 15078.
Pdhk4	Commander, R., Wei, C., Sharma, A., Mouw, J.K., Burton, L.J., Summerbell, E., Mahboubi, D., Peterson, R.J., Konen, J., and Zhou, W., et al. (2020). Subpopulation targeting of pyruvate dehydrogenase and GLUT1 decouples metabolic heterogeneity during collective cancer cell invasion. Nat Commun 11, 1533.

Cd44	Yang, C., Cao, M., Liu, Y., He, Y., Du, Y., Zhang, G., and Gao, F. (2019). Inducible formation of leader cells driven by CD44 switching gives rise to collective invasion and metastases in luminal breast carcinomas. Oncogene 38, 7113-7132.
Amigo2	Sonzogni, O., Haynes, J., Seifried, L.A., Kamel, Y.M., Huang, K., BeGora, M.D., Yeung, F.A., Robert-Tissot, C., Heng, Y.J., and Yuan, X., et al. (2018). Reporters to mark and eliminate basal or luminal epithelial cells in culture and in vivo. PLoS Biol 16, e2004049.
Myo10<br>Jag1<br>Fn1<br>Arl4c<br>Ripk4<br>Rnf182<br>Ctcfl<br>Srpx	Summerbell, E.R., Mouw, J.K., Bell, J.S.K., Knippler, C.M., Pedro, B., Arnst, J.L., Khatib, T.O., Commander, R., Barwick, B.G., and Konen, J., et al. (2020). Epigenetically heterogeneous tumor cells direct collective invasion through filopodia-driven fibronectin micropatterning. Sci Adv 6, eaaz6197.

SUPPLEMENTARY FIGURES

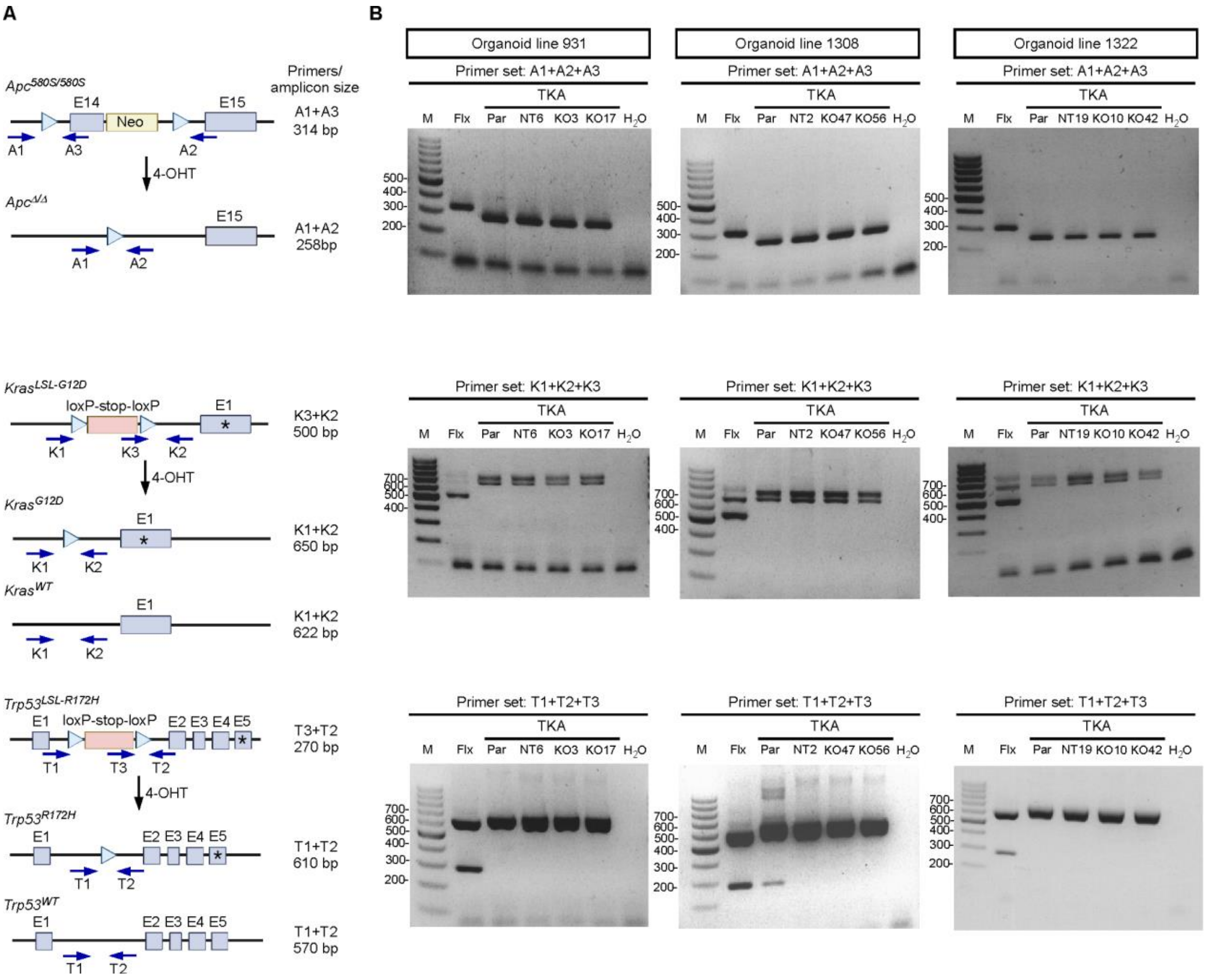

Supplementary figure 1. Genotype confirmation of organoid lines at the *Apc*, *Kras*, and *Trp53* loci. A The schemes depict the structures of the floxed *Apc*, *Kras* and *Trp53* loci, the resultant loci after Cre-mediated recombination, and the wild-type (WT) alleles where applicable. Blue boxes: exons (E) with locus-specific numbering; exons in *Kras* and *Trp53* carrying the *Kras*^{G12D} and *Trp53*^{R172H} mutations, respectively, are marked with asterisks. Yellow box: neomycin (Neo) resistance gene. Light red boxes: transcriptional/translational stop cassettes preventing expression of *Kras*^{G12D} and *Trp53*^{R172H}. Blue triangles: loxP elements. Dark blue arrows: positions of PCR primers. 4-OHT: 4-hydroxytamoxifen. The sizes of PCR amplicons arising from primer combinations selective for floxed, recombined and WT alleles are shown next to the genomic schemes. Created with BioRender.com. **B** Results of genotyping PCRs with locus-specific combinations of PCR primers (A: *Apc*; K: *Kras*; T: *Trp53*; see panel A) and genomic DNA collected from floxed (Flx) and TKA organoid lines as indicated. Floxed organoids and parental (par) TKA-organoids as well as their clonal derivatives exposed to non-targeting sgRNAs (NT) have WT *Sox11* genes. Organoid lines with a "KO" prefix in their names have biallelic deletions in their *Sox11* genes (see Supplementary figure 7). No template PCR controls received corresponding volumes of double distilled water (H_2O) instead of genomic DNA. M: size marker; lengths of DNA fragments are given in bp.

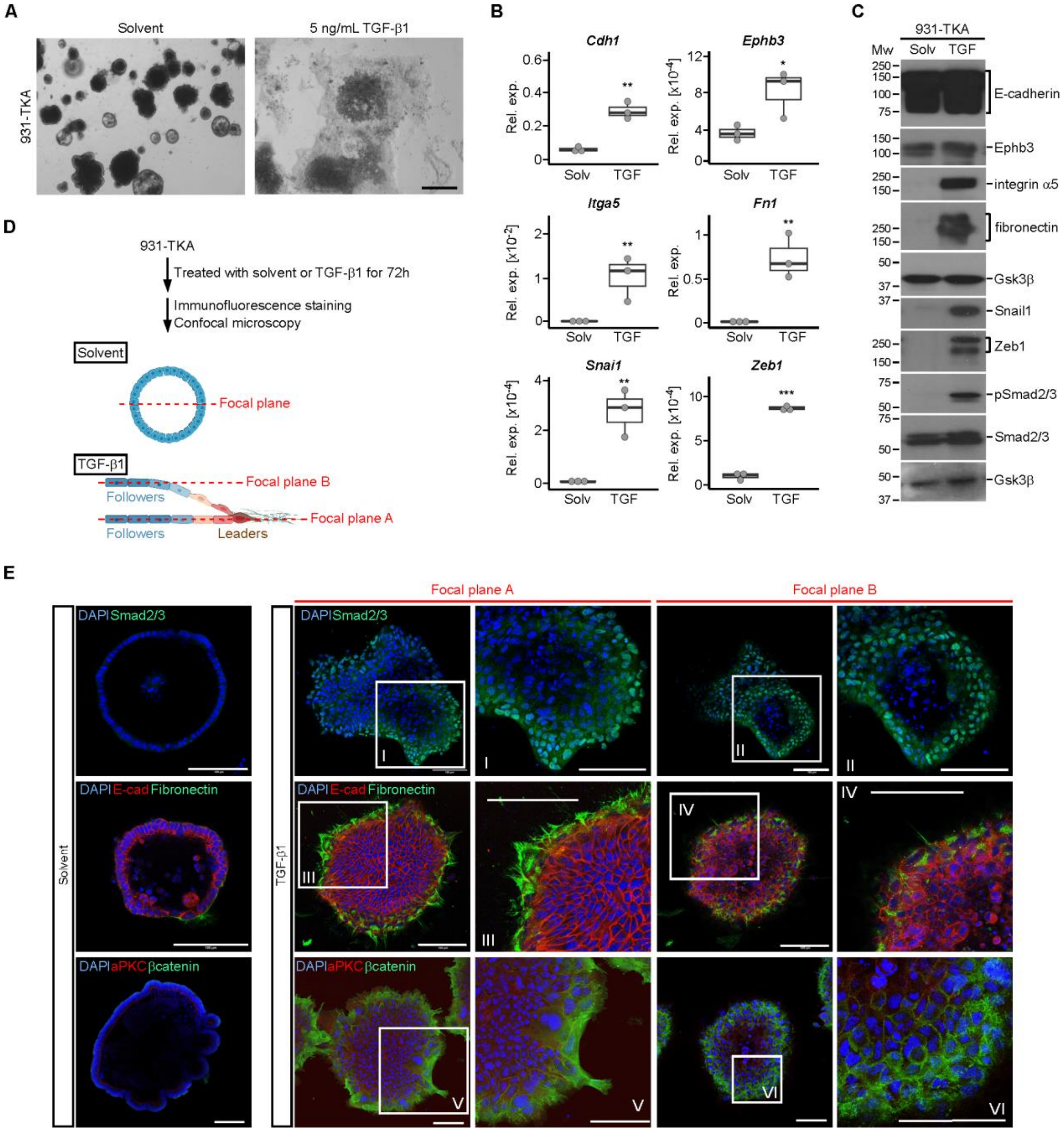

Supplementary figure 2. TGF-β1-treated TKA-organoids display partial EMT states and cellular heterogeneity. A Whole-mount phase contrast microscopy of 931-TKA organoids treated with solvent or TGF-β1 for 72 h. Scale bar: 100 µm. Representative images from one of three independent biological replicates are shown. **B** RNA expression of epithelial (*Cdh1*, *Ephb3*) and mesenchymal markers (*Itga5*, *Fn1*, *Snai1*, *Zeb1*) in 931-TKA organoids. Organoids were seeded in 3 mg/ml Matrigel and treated with solvent or TGF-β1 for 72 h, followed by RNA collection and cDNA synthesis. Relative expression levels (rel. exp.) were determined by qRT-PCR and normalization to *Eef1a1* expression (n=3). The box plots show the 26th to 75th percentiles of the data and the median. Dots represent results of individual experiments. The statistical analyses were performed using one-way ANOVA, *: *p-value* < 0.05; **: *p*-value < 0.01; ***: *p*-value < 0.001. **C** Protein expression levels of epithelial markers (E-cadherin, Ephb3) and mesenchymal markers (integrin α5, fibronectin, Snail1, Zeb1) 931-TKA organoids treated with solvent or TGF-β1 for 72 h were determined by western blot analyses. Phosphorylated Smad2/3 (pSmad2/3) and total Smad2/3 amounts were analyzed to show TGF-β pathway activation. Detection

of Gsk3β served to control equal loading. Mw: molecular weight standards in kDa. Images are representative examples from one of three biological replicates. **D** Schematic representation of the experimental strategy used for whole-mount immunofluorescence staining and confocal microscopy. Positions of focal planes examined are shown in the schemes of 931-TKA organoids treated with solvent or TGF-β1 for 72 h. Created with BioRender.com. **E** 931-TKA organoids were seeded in 3 mg/ml Matrigel and treated with solvent or TGF-β1 for 72 h, followed by whole-mount immunofluorescence staining of the antigens indicated. Nuclei were counterstained with DAPI. The boxed areas labeled with Roman numerals are shown at higher magnification. The positions of the focal planes are depicted in panel D. Scale bar: 100 µm.

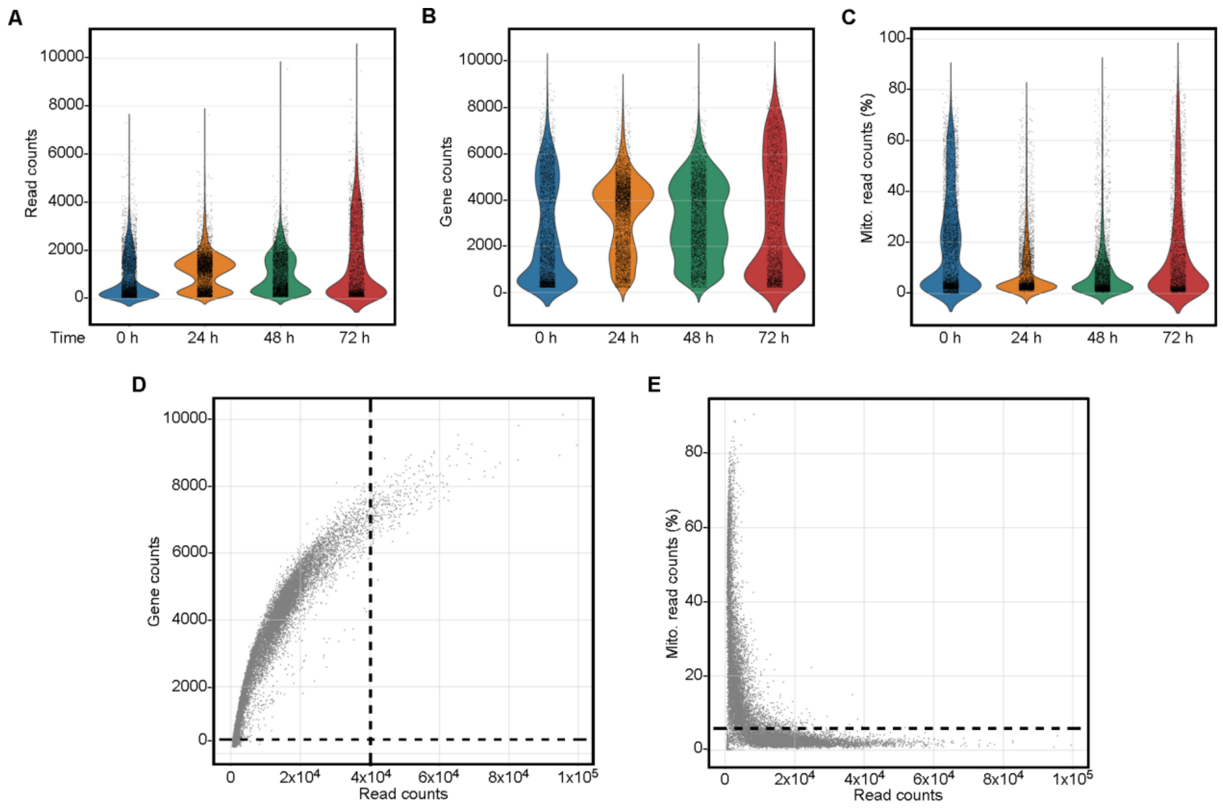

F

Duration of TGF-β1 treatment	Number of cells per time point
0 h	1222
24 h	2924
48 h	1886
72 h	831

UMAP Cluster	Number of cells per cluster
0	1901
1	1139
2	1001
3	916
4	592
5	436
6	373
7	358
8	147

Supplementary figure 3. Quality control measures of scRNA-seq raw data. A-C Pre-filtering summary data for read counts, gene counts, and percentages of mitochondrial read counts in scRNA-seq raw data from 931-TKA organoid cells resolved by TGF-β1 treatment times. **D, E** Gene counts mapped to the mouse genome (D) and percentages of mitochondrial read counts (E) relative to the total read counts in scRNA-seq raw data. The count matrices for individual organoid cells were generated by aligning raw reads to the mouse reference genome. Genes detected in fewer than 3 cells, cells expressing fewer than 200 genes, with less than 2000 read counts, with more than 40 000 read counts, and percentages of mitochondrial read counts exceeding 4% were excluded from the subsequent analyses. The dashed lines show the indicated thresholds. **F** The number of 931-TKA organoid cells available for bioinformatic analyses after scRNA-seq quality control and filtering steps were listed according to duration of TGF-β1 treatment and assignment to the different UMAP clusters.

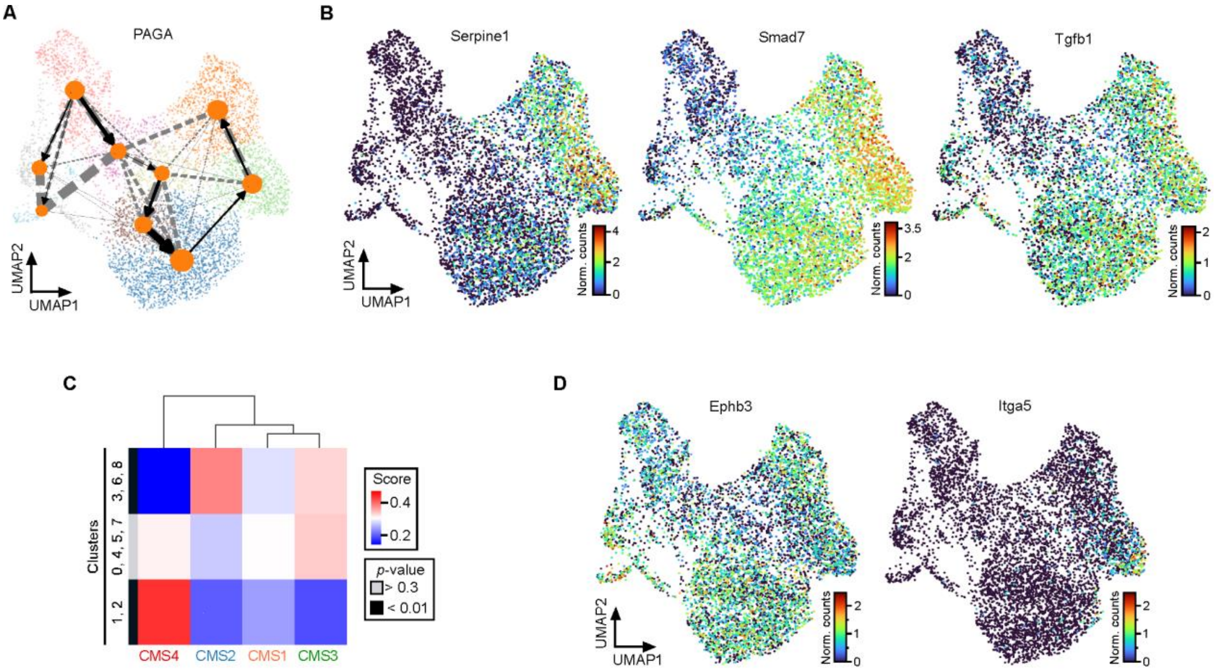

Supplementary figure 4. TGF-β1 induced gene expression and CMS transitions of TKA-organoids. A Partition-based graph abstraction (PAGA) of scRNA-seq results from 931-TKA organoids treated with TGF-β1 for 0, 24, 48, and 72 h. PAGA reconstructs potential lineage trajectories based on RNA velocity maps. The black arrows depict potential developmental routes and the dashed lines show the connectivity of the clusters. The thickness of the lines represents the strength of connectivity between the clusters. **B** UMAPs visualizing expression levels of TGF-β pathway regulated genes in organoid cells treated with TGF-β1 for 0, 24, 48, and 72 h. **C** The MmCMS package was used to predict the consensus molecular subtype (CMS) score of control and TGFβ1-treated organoid cells using pseudobulk transcriptomes aggregated from scRNA-seq results of individual cells in the clusters shown. The red-blue color scale reflects the CMS enrichment score. Black and grey labels denote statistical significance (black: *p*-value < 0.01; grey: not significant, *p*-value > 0.3). **D** UMAPs visualizing expression levels of additional examples for epithelial and mesenchymal markers in TGF-β1-treated organoid cells. In (B) and (D) the color codes represent the normalized counts (Norm. counts) of the indicated genes.

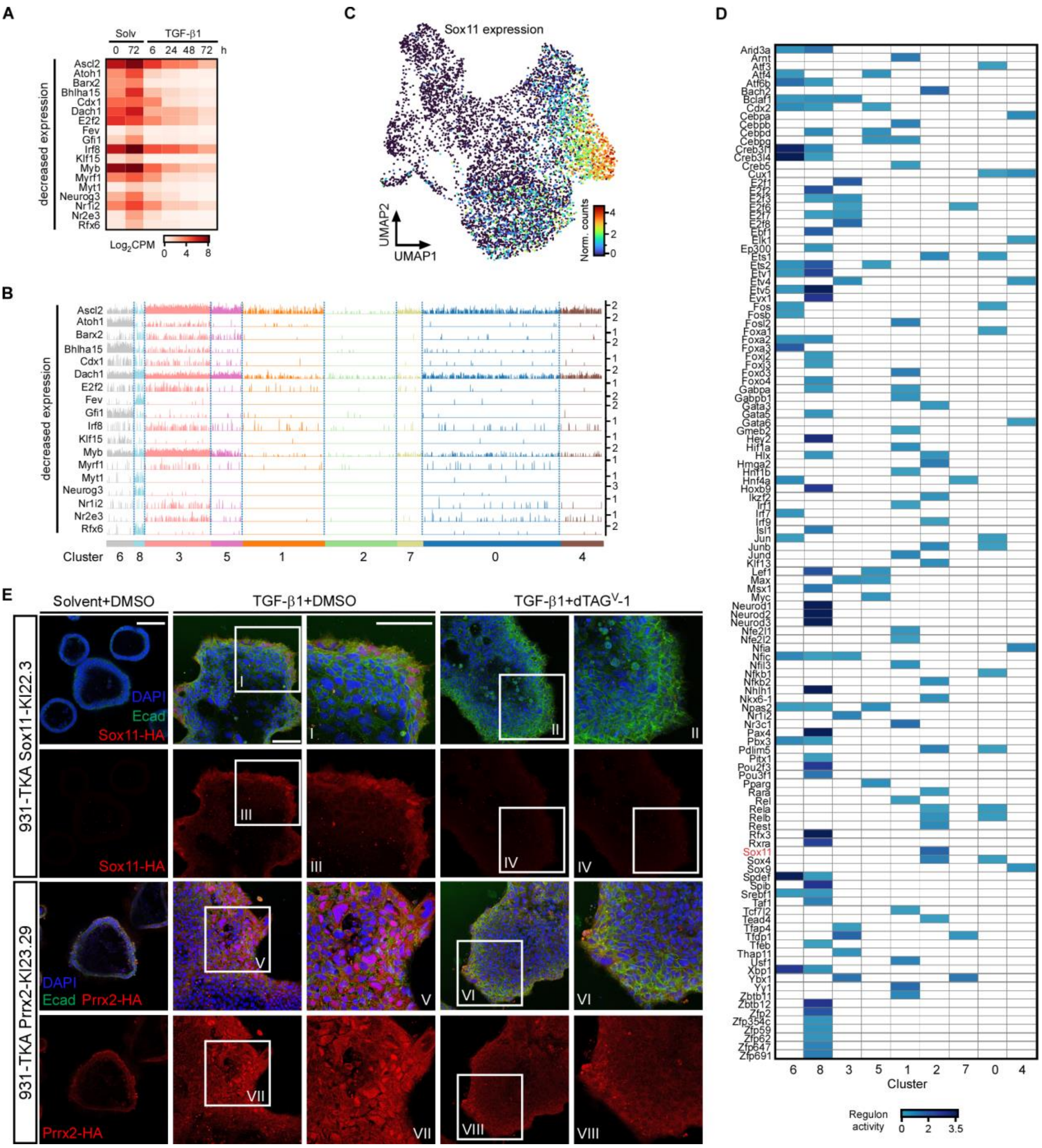

Supplementary figure 5. Transcription factor expression, regulon activity, and differential localization of Sox11-HA and Prrx2-HA in TGF-β1-treated TKA-organoids. A Heatmap displaying TF genes with decreasing expression in combined, time-resolved bulk RNA-seq data from solvent (Solv) or TGF-β1-treated 931-TKA and 947-TKA organoids. All murine TFs with $\log_2$ fold changes < -4 at least at one time point of TGF-β1 stimulation were selected for display. Samples treated with TGF-β1 for 6 h and 24 h were compared to samples harvested at the beginning of the treatment (solvent 0 h). Samples treated with TGF-β1 for 48 h and 72 h were compared to samples kept in solvent for 72 h. Normalized $\log_2$CPM values were used as gene expression units. **B** Trackplots showing cluster-wise expression of the same TF genes as in (A) in scRNA-seq data from solvent or TGF-β1-treated 931-TKA organoid cells. Each peak represents the normalized count for a given gene in a single organoid cell. **C** UMAP visualization of *Sox11* expression in scRNA-seq data. The color code represents the normalized counts of *Sox11* transcripts. **D** Cluster-specific TF activities in 931-TKA organoid cells were inferred by SCENIC

and are displayed as regulon activity. **E** 931-TKA clones Sox11-KI22.3 and Prrx2-KI23.79 were seeded in 3 mg/ml Matrigel and treated with solvent or TGF-β1 for 72 h. Two hours prior to the experimental endpoint, organoids additionally received DMSO or 500 nM dTAG^{V}-1. Subsequently, organoids were processed for whole-mount immunofluorescence staining of the indicated antigens and confocal microscopy. Nuclei were counterstained with DAPI. Boxed areas with Roman numerals are also shown at higher magnification. Images are representative examples from one of at least three biological replicates (n≥3). Scale bars: 100 µm.

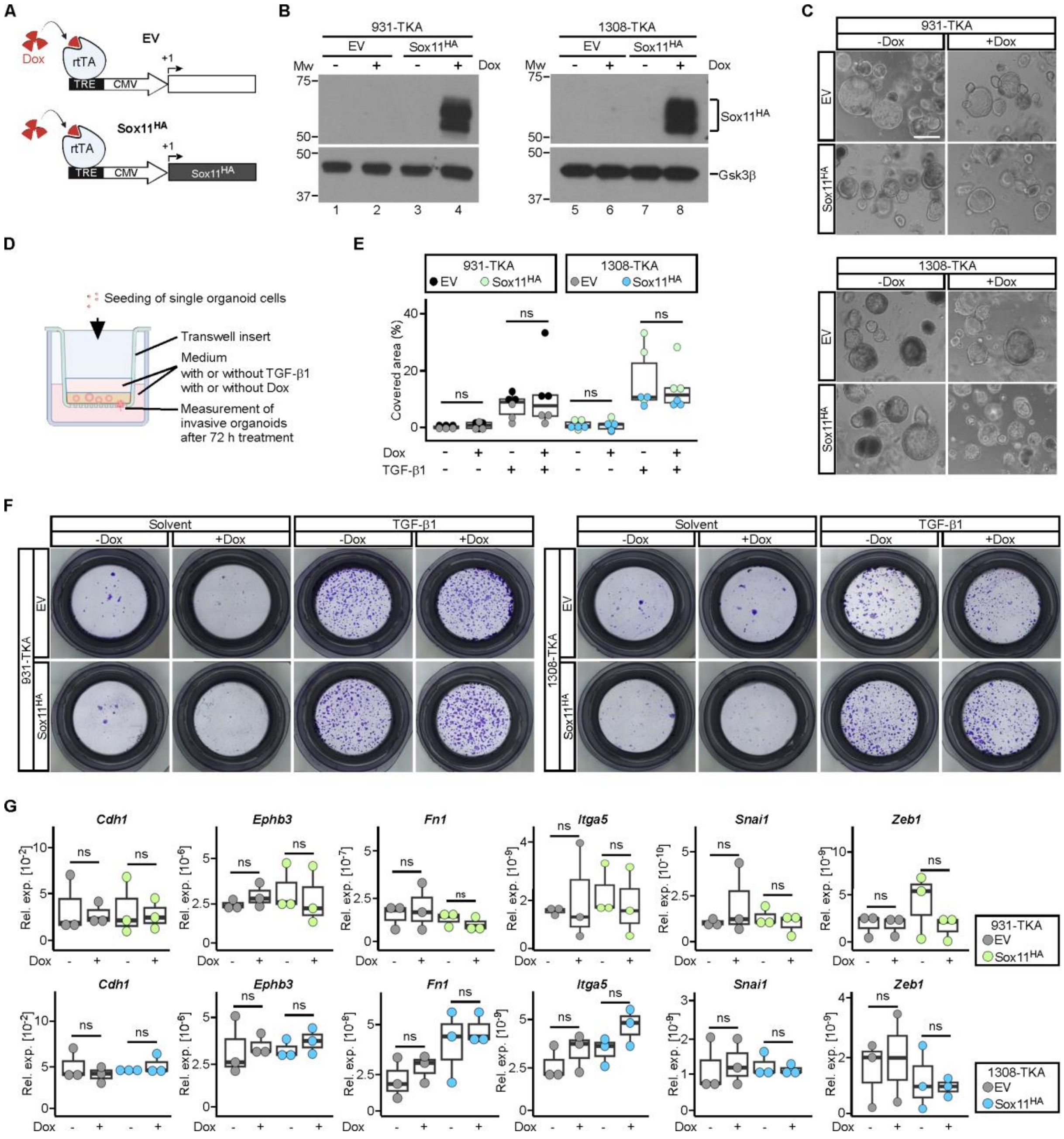

Supplementary figure 6. Overexpression of Sox11 does not affect organoid morphology and epithelial/mesenchymal marker gene expression. A Schematic depiction of the retroviral vector system for doxycycline (Dox)-inducible overexpression Sox11 tagged with an HA-epitope (Sox11^{HA}). Expression of Sox11^{HA} is driven by the tight promoter (P_{tight}) which is controlled by the reverse tetracycline-dependent transcriptional activator (rtTA). The empty vector (EV) served as control. Created with BioRender.com. **B** Western blot analyses of Sox11^{HA} expression in 931-TKA and 1308-TKA organoids transduced with EV and Sox11^{HA} constructs. Organoids were treated with Dox for 72 h. Detection of Gsk3β was employed to control equal loading. Mw: molecular weight standards in kDa. Representative results from one of three biological replicates are shown. **C** Whole-mount phase contrast microscopy of 931-TKA and 1308-TKA organoid lines transduced with EV and Sox11^{HA} constructs and treated with Dox for 72 h. Scale bar: 100 µm. Representative pictures from one of three biological replicates are shown. **D** Schematic depiction of the transwell invasion assay. After enzymatic dissociation of organoids, 1x10^5 single cells were mixed with 3 mg/ml Matrigel and seeded into the

transwell inserts. Once organoid cells had formed cystic structures, they were treated with the indicated combinations of solvent, TGF-β1, and Dox for 72 h. Invasive organoid cells that had passed through the Matrigel layer and crossed the transwell bottom membrane were visualized by crystal violet staining. Areas of transwell membranes covered by invaded organoid cells were quantified by ImageJ. Created with BioRender.com. **E** Quantification of transwell invasion assays with 931-TKA and 1308-TKA organoids transduced with EV and Sox11^{HA} constructs. Areas of transwell membranes covered by invaded organoid cells as exemplarily shown in (F) were measured by ImageJ (n=3). The box plots show the 26th to 75th percentiles of the data, and the median. Dots represent results of individual measurements. Statistical significance was assessed by one-way ANOVA; ns: not significant. **F** Representative images of transwell invasion assays with 931-TKA and 1308-TKA organoid lines transduced with EV and Sox11^{HA} constructs. The assay was conducted exactly as described in panel D. **G** Expression of epithelial (*Cdh1*, *Ephb3*) and mesenchymal markers (*Itga5*, *Fn1*, *Snai1*, *Zeb1*) in 931-TKA and 1308-TKA organoids transduced with EV and Sox11^{HA} constructs. Organoids were seeded in 3 mg/ml Matrigel and treated with or without Dox for 72 h, followed by RNA collection and cDNA synthesis. Relative expression (rel. exp.) levels of RNA levels were determined by qRT-PCR and normalization to *Eef1a1* expression (n=3). The box plots show the 26th to 75th percentiles of the data, and the median. Dots represent results of individual measurements. Statistical significance was assessed by one-way ANOVA; ns: not significant.

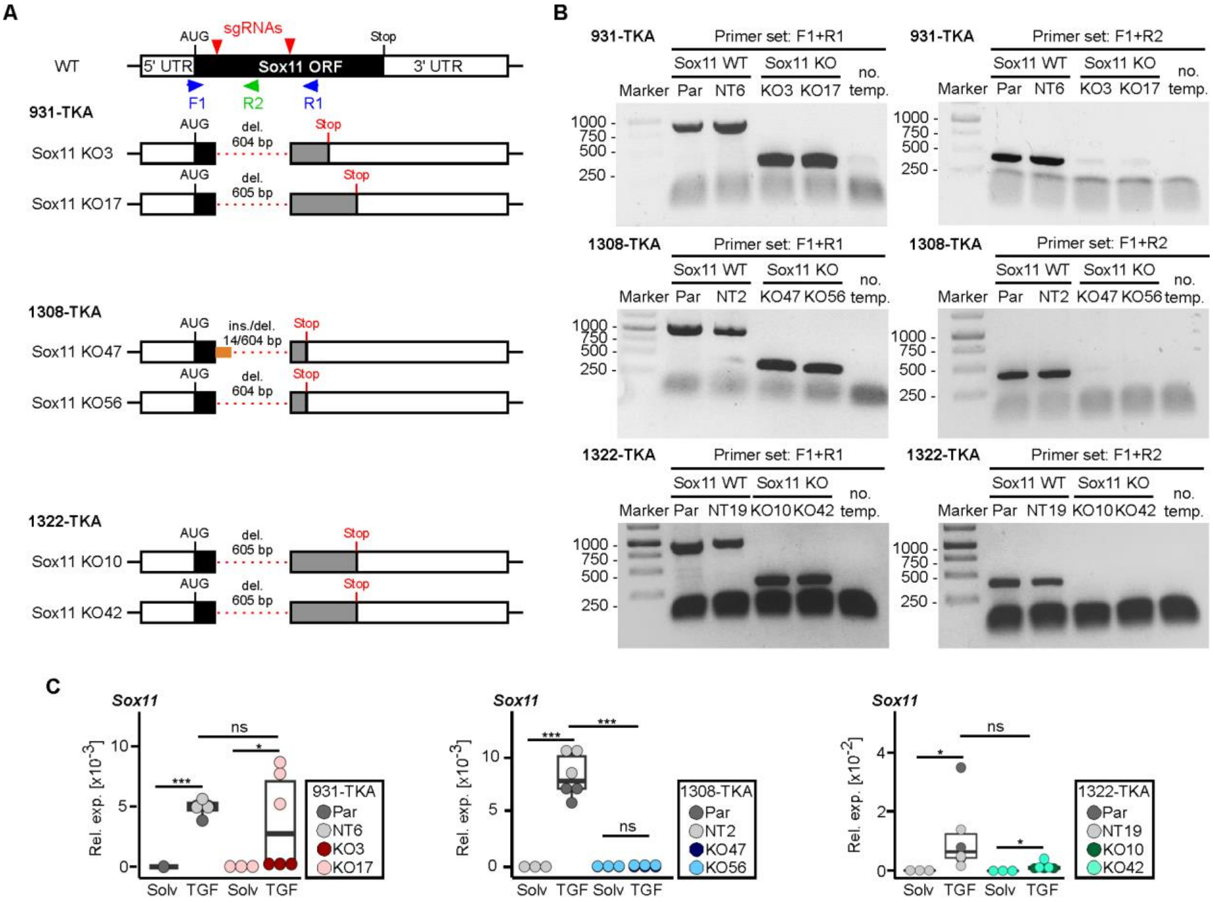

Supplementary figure 7. CRISPR/Cas9-mediated knock-out of the *Sox11* gene. A Strategy to inactivate *Sox11* and structures of the WT and knock-out (KO) alleles in organoid lines 931-TKA, 1308-TKA, and 1322-TKA. The black boxes represent the *Sox11* open reading frame (ORF) or the remainder thereof in the KO alleles. Positions of start (AUG) and stop codons are given. White boxes show untranslated regions (UTR). Grey boxes specify newly translated regions and corresponding stop codons upon deletion-induced frame shifts. The red arrowheads indicate sgRNA target positions. The primer set F1+R1 was used for PCR-based detection of CRISPR/Cas9-mediated deletions in *Sox11*. Primer set F1+R2 was employed to ascertain absence of WT alleles and confirmation of biallelic deletions in *Sox11*. The red dashed lines denote deleted parts of the *Sox11* gene. The number of deleted (del.) bp is shown. The orange box represents a 14 bp insertion (ins.) in one of the alleles of clone 1308-TKA Sox11 KO47. **B** Results of genotyping PCRs with genomic DNA collected from the indicated parental (par) TKA organoid lines and clonally derived organoids that had been treated with non-targeting (NT) or *Sox11*-targeting sgRNAs. PCRs were performed with primer sets F1+R1 and F1+R2 as depicted. No template controls (no. temp.) received corresponding volumes of water instead of genomic DNA. **C** *Sox11* expression in *Sox11* WT and KO organoids. Organoids were seeded in 3 mg/ml Matrigel and treated with solvent or TGF-β1 for 72 h, followed by RNA collection and cDNA synthesis. Relative expression (rel. exp.) levels of *Sox11* were determined by qRT-PCR and normalization to *Eef1a1* expression. The box plots show the 26th to 75th percentiles of the data and the median. Dots represent results of individual experiments (n=3). Statistical analyses were performed using one-way ANOVA; ns: not significant; *: *p-value* < 0.05; ***: *p*-value < 0.001. Solv: solvent; TGF: TGF-β1.

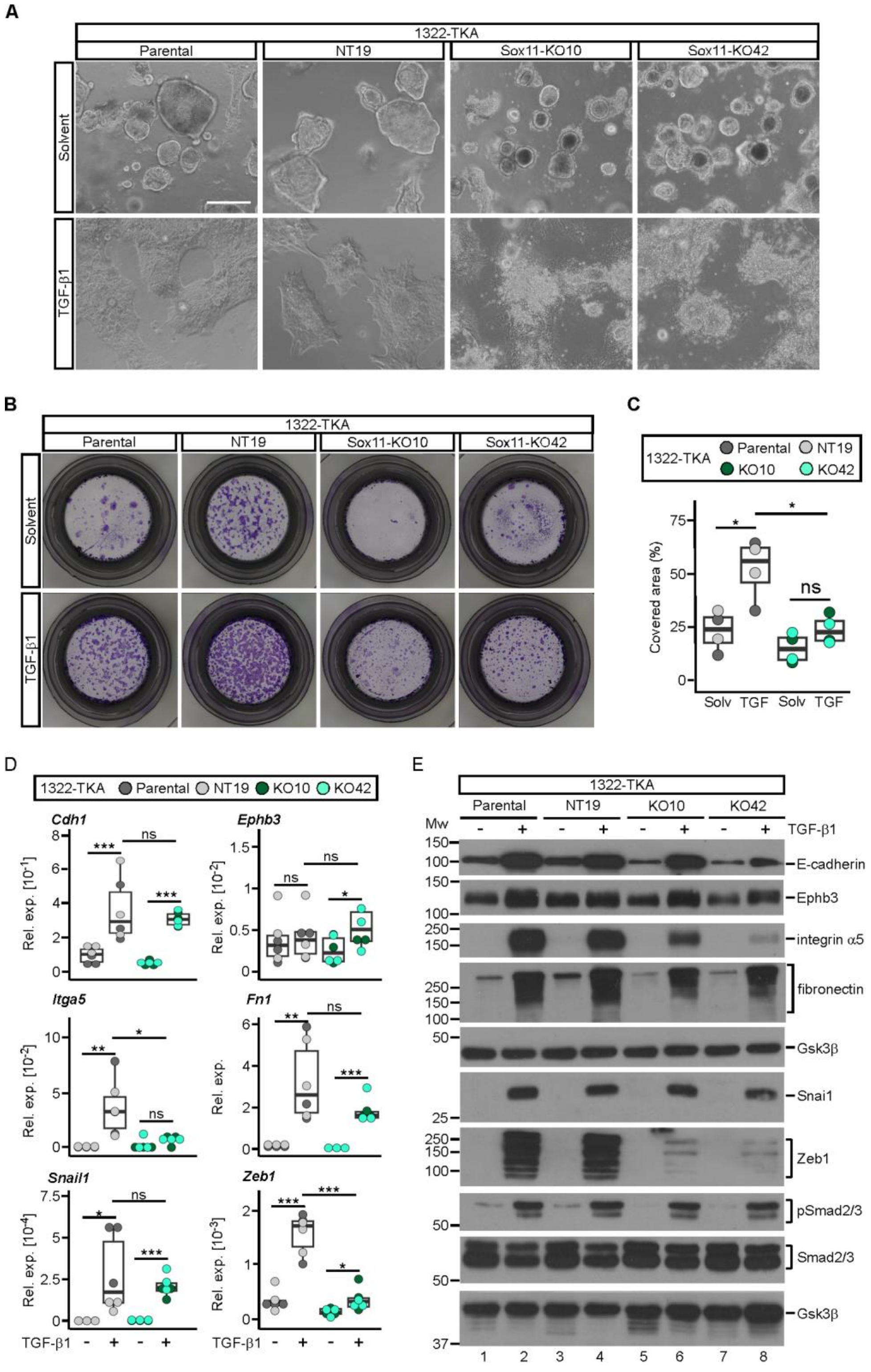

Supplementary figure 8. Inactivation of *Sox11* reduces collective invasion and pEMT in 1322-TKA organoids. A Whole-mount phase contrast microscopy of *Sox11* WT (parental; NT19) and *Sox11* mutant (KO10; KO42) organoids (line 1322-TKA) treated with solvent or TGF-β1 for 72 h. Scale bar: 100 µm. Representative pictures from one of three independent biological replicates are shown. **B** Representative images of transwell invasion assays performed with *Sox11* WT (parental; NT19) and *Sox11* mutant (KO10; KO42) organoids (line 1322-TKA) as indicated. Organoids were seeded in 3 mg/ml Matrigel in the upper chambers of transwell inserts and treated with TGF-β1 for 72h. Invasive organoid cells that had passed through the Matrigel layer and crossed the transwell bottom membrane

were visualized by crystal violet staining. **C** Quantification of transwell assays with *Sox11* WT (parental; NT19) and *Sox11* mutant (KO10; KO42) organoids (line 1322-TKA). Areas of transwell membranes covered by invaded organoid cells as exemplarily shown in (B) were measured by ImageJ (n=3). Statistical significance was assessed by one-way ANOVA, ns: not significant; *: p-value < 0.05. **D** RNA expression levels of epithelial (*Cdh1*, *Ephb3*) and mesenchymal markers (*Itga5*, *Fn1*, *Snai1*, *Zeb1*) in the indicated *Sox11* WT and mutant organoid lines treated with solvent or TGF-β1 for 72 h were determined by qRT-PCR and normalized to those of *Eef1a1* (n=3). The box plots show the 26th to 75th percentiles of the data and the median. Dots represent results of individual measurements. Rel. exp.: relative expression. One-way ANOVA; ns: not significant, *: p-value < 0.05; **: p-value < 0.01; ***: p-value < 0.001. **E** Protein expression levels of epithelial markers (E-cadherin, Ephb3) and mesenchymal markers (integrin α5, fibronectin, Snail1, Zeb1) in the indicated *Sox11* WT (parental; NT19) and *Sox11* mutant (KO10; KO42) organoids (line 1322-TKA) treated with solvent or TGF-β1 for 72 h were determined by western blot analyses. Phosphorylated Smad2/3 (pSmad2/3) and total Smad2/3 amounts were analyzed to show TGF-β pathway activation. Detection of Gsk3β served to control equal loading. Mw: molecular weight standards in kDa. Images are representative examples from one of three independent biological replicates.

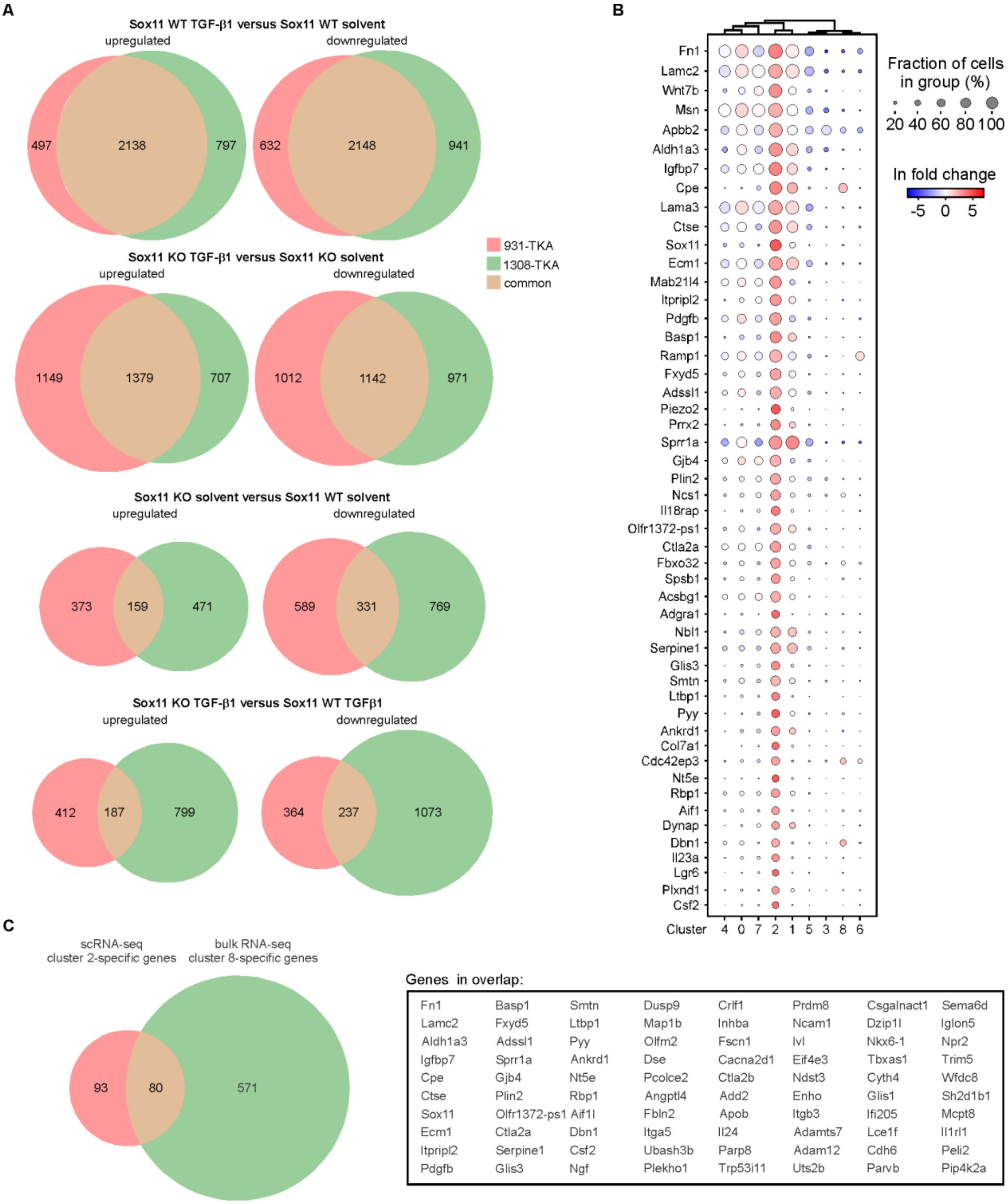

Fn1	Basp1	Smtn	Dusp9	Crlf1	Prdm8	Csgalnact1	Sema6d
Lamc2	Fxyd5	Ltbp1	Map1b	Inhba	Ncam1	Dzip1l	Iglon5
Aldh1a3	Adssl1	Pyy	Olfm2	Fscn1	Ivl	Nkx6-1	Npr2
Igfbp7	Sprr1a	Ankrd1	Dse	Cacna2d1	Eif4e3	Tbxas1	Trim5
Cpe	Gjb4	Nt5e	Pcolce2	Ctla2b	Ndst3	Cyth4	Wfdc8
Ctse	Plin2	Rbp1	Angptl4	Add2	Enho	Glis1	Sh2d1b1
Sox11	Olfr1372-ps1	Aif1l	Fbln2	Apob	Itgb3	Ifi205	Mcpt8
Ecm1	Ctla2a	Dbn1	Itga5	Il24	Adamts7	Lce1f	Il1rl1
Itpripl2	Serpine1	Csf2	Ubash3b	Parp8	Adam12	Cdh6	Peli2
Pdgfb	Glis3	Ngf	Plekho1	Trp53i11	Uts2b	Parvb	Pip4k2a

Supplementary figure 9. Characterization of gene expression profiles of *Sox11* WT and mutant TKA-organoids by bulk RNA-seq. A Venn diagrams showing the numbers of DEGs unique and common to 931-TKA and 1308-TKA organoids sorted by genotypes and treatments as indicated. DEGs included in the comparisons were up- or downregulated with absolute values of $\log_2$ fold changes > 1 and adjusted *p*-values < 0.05. **B** The dotplot function in the SCANPY package was used to predict genes expressed at comparatively higher levels in pEMT^{high}/presumptive leader cells from scRNA-seq cluster 2. The size of each dot corresponds to the fraction of cells within a cluster which express a given gene. The color code indicates the ln fold change of gene expression in a specific cluster compared to the mean expression across all other clusters. Only the top 50 genes are shown. **C** Comparison of genes

in scRNA-seq cluster 2 and bulk RNA-seq cluster 8. The 80 genes common to both groups are listed beneath the Venn diagram. Genes were selected for the analyses based on ln fold changes > 2 and adjusted p-values < 0.05 (scRNA-seq) and absolute values of $\log_2$ fold changes > 1 and adjusted p-values < 0.05 (bulk RNA-seq).

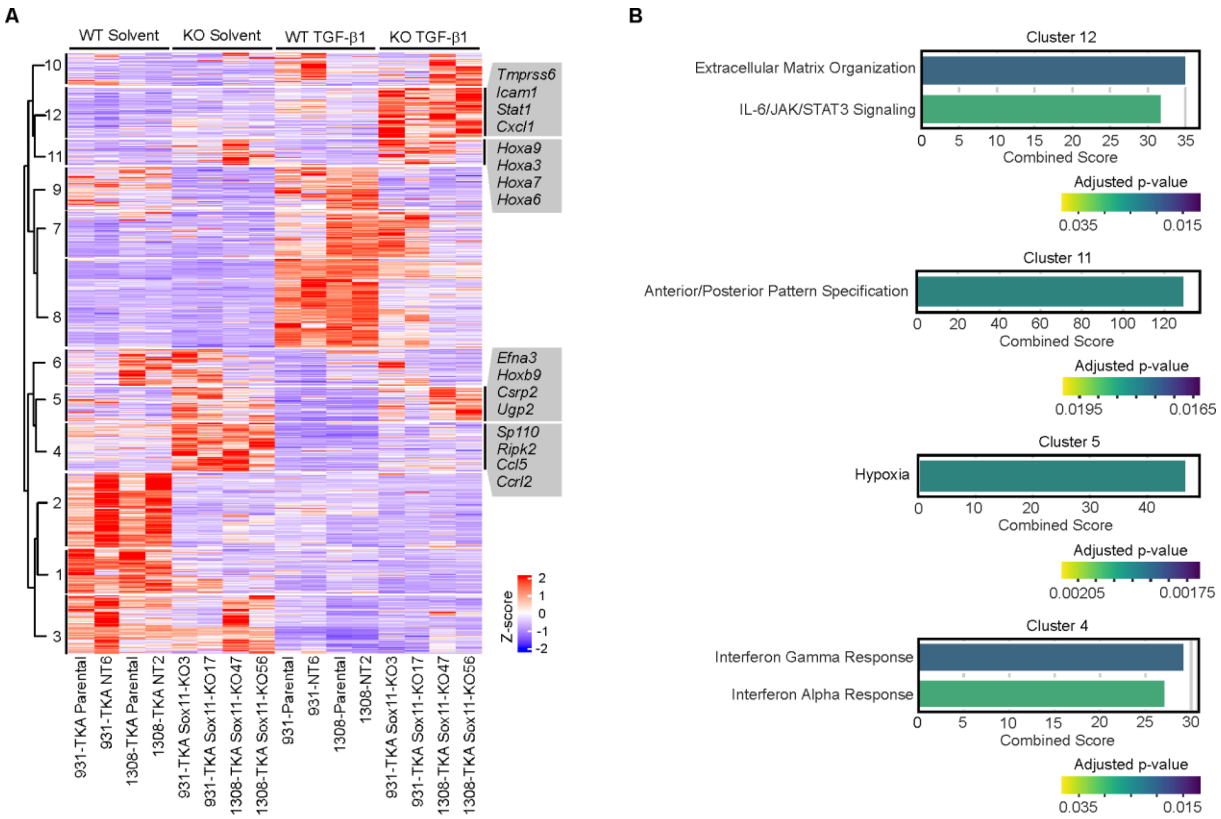

Supplementary figure 10. Pathway analyses for genes aberrantly upregulated in *Sox11* mutant organoids in the presence or absence of TGF-β1. A Heatmap showing DEGs in the indicated *Sox11* WT and *Sox11* mutant organoid lines treated with solvent or TGF-β1 for 72 h. Genes with absolute values of $\log_2$ fold changes of expression > 1 and adjusted *p*-values < 0.05 in any condition when comparing *Sox11* WT and KO organoid lines were extracted from the bulk RNA-seq results. Gene expression levels in counts per million (CPM) were then normalized by z-score transformation, sorted with a pre-determined number of 12 clusters, and visualized in the heatmap. Selected genes from clusters 4, 5, 11, and 12 are shown next to the heatmap. The color code indicates z-scores of the genes. **C** DEGs from clusters 4, 5, 11, and 12 in panel A were examined for enrichment of gene expression signatures from the MSigDB Hallmark and Reactomes datasets and the GO collection Biological Process. Results of pathway analyses are given as combined scores based on adjusted *p*-values and odds ratios which reflect the fraction of genes from a given gene set showing enrichment among DEGs versus the total number of genes in the respective set. Color codes indicate adjusted *p*-values with Fisher's exact test.

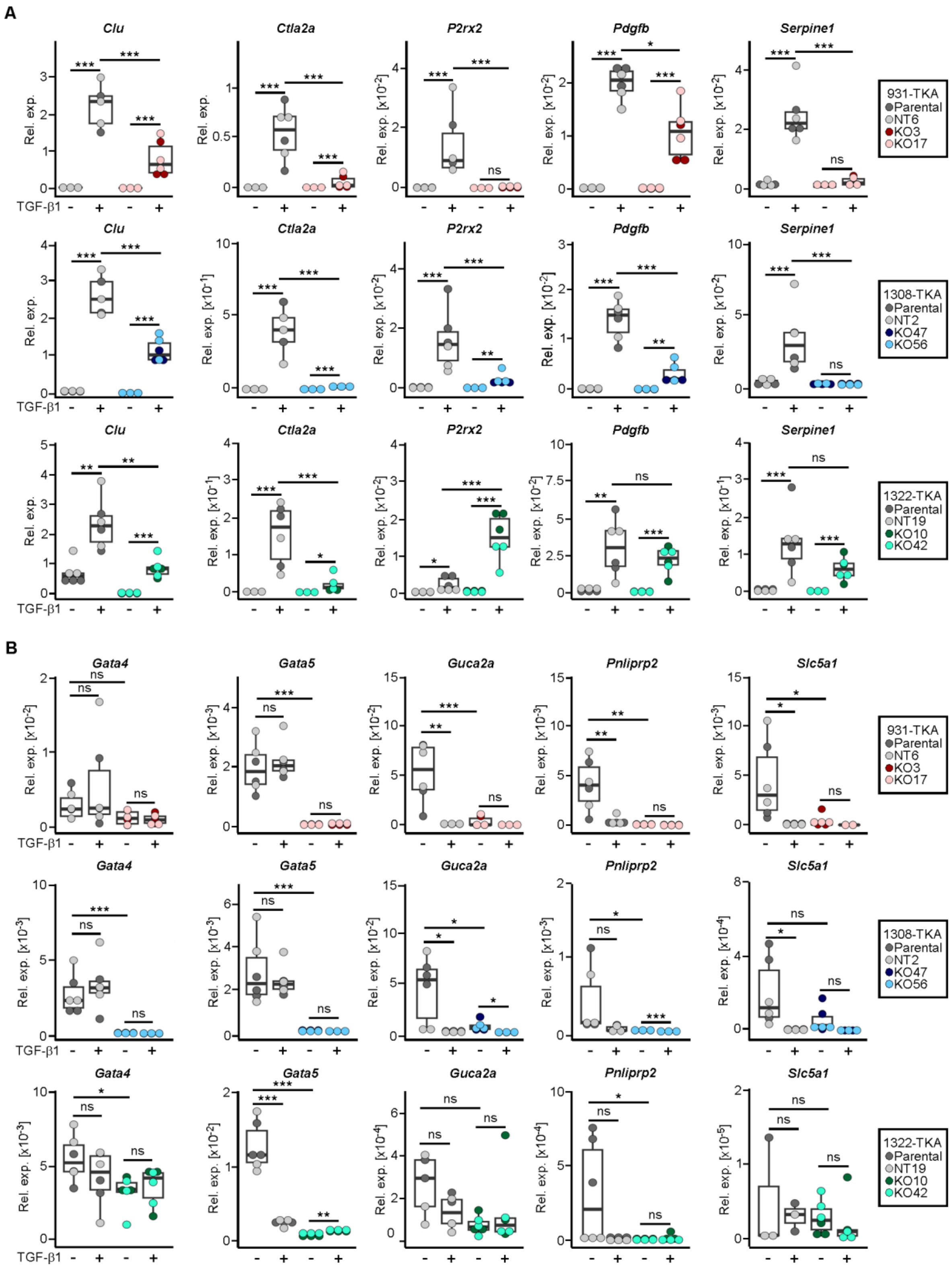

Supplementary figure 11. Validation of bulk RNA-seq results. A, B 931-TKA, 1308-TKA, and 1322-TKA organoids with WT (parental; NT clones) and inactivated *Sox11* genes (KO clones) were treated with solvent or TGF-β1 for 72 h, and relative expression (rel. exp.) levels of selected genes from (A) bulk RNA-seq cluster 8, and (B) from clusters 1, 2, and 9 (*Gata5*) were determined by qRT-PCR and normalization to *Eef1a1* expression (n=3). The box plots show the 26th to 75th percentiles of the data, and the median. Dots represent results of individual measurements. The statistical analyses were

performed using one-way ANOVA; ns: not significant; *: p-value < 0.05; **: p-value < 0.01; ***: p-value < 0.001.

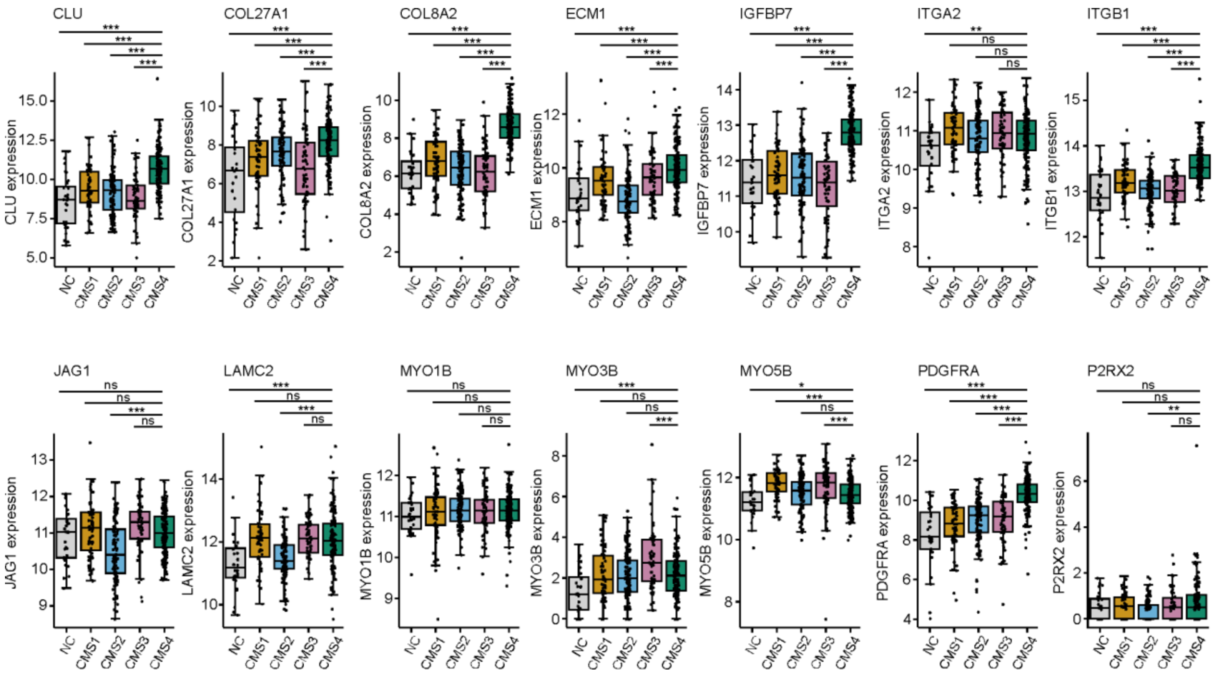

Supplementary figure 12. Expression of human orthologues of *Sox11*-dependent genes in CRC samples. Box plots visualizing expression of the genes indicated in CMS-stratified transcriptomes of COAD and READ primary tumors from the TCGA database. Each black dot shows an individual sample. The box plots show the 26th to 75th percentiles of the data and the median. Statistical analyses were performed using the Mann-Whitney U test; ns: not significant; **: *p*-value < 0.01; ***: *p*-value < 0.001. NC: not classifiable.

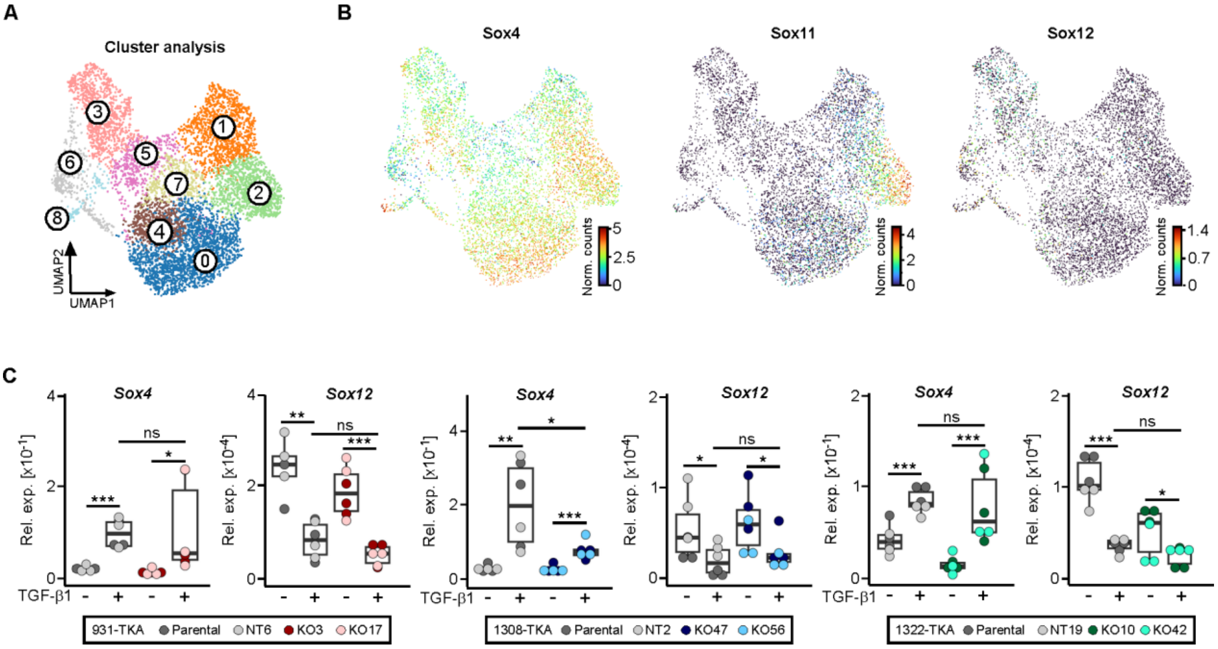

Supplementary figure 13. Expression of Sox family members in *Sox11* WT and mutant organoid lines. A Unsupervised clustering and UMAP visualization of 931-TKA organoid cells upon TGF-β1 treatment for 0, 24, 48, and 72 h. **B** UMAPs visualizing expression levels of SoxC family members *Sox4*, *Sox11*, and *Sox12*. **C** *Sox4* and *Sox12* expression in *Sox11* WT (parental, NT clones) and mutant (KO clones) organoids (lines 931-TKA, 1308-TKA, and 1322-TKA) treated with solvent or TGF-β1 for 72 h. Relative expression (rel. exp.) levels of the genes indicated were determined by qRT-PCR and normalization to *Eef1a1* expression (n=3). The box plots show the 26th to 75th percentiles of the data, and the median. Dots represent results of individual measurements. The statistical analyses were performed using one-way ANOVA; ns: not significant (p-value > 0.05), *: p-value < 0.05; **: p-value < 0.01;***: p-value < 0.001.

SUPPLEMENTARY REFERENCES:

1. Flum M, Dicks S, Teng Y-H, Schrempp M, Nyström A, Boerries M et al. Canonical TGFβ signaling induces collective invasion in colorectal carcinogenesis through a Snail1- and Zeb1-independent partial EMT. Oncogene 2022; 41:1492–506.
2. Artegiani B, Hendriks D, Beumer J, Kok R, Zheng X, Joore I et al. Fast and efficient generation of knock-in human organoids using homology-independent CRISPR-Cas9 precision genome editing. Nat Cell Biol 2020; 22:321–31.
3. Schmid-Burgk JL, Höning K, Ebert TS, Hornung V. CRISPaint allows modular base-specific gene tagging using a ligase-4-dependent mechanism. Nat Commun 2016; 7:12338.
4. Rönsch K, Jägle S, Rose K, Seidl M, Baumgartner F, Freihen V et al. SNAIL1 combines competitive displacement of ASCL2 and epigenetic mechanisms to rapidly silence the EPHB3 tumor suppressor in colorectal cancer. Mol Oncol 2015; 9:335–54.
5. Antón-García P, Haghighi EB, Rose K, Vladimirov G, Boerries M, Hecht A. TGFβ1-Induced EMT in the MCF10A Mammary Epithelial Cell Line Model Is Executed Independently of SNAIL1 and ZEB1 but Relies on JUNB-Coordinated Transcriptional Regulation. Cancers (Basel) 2023; 15.
6. The Galaxy Community. The Galaxy platform for accessible, reproducible, and collaborative data analyses: 2024 update. Nucleic Acids Res 2024; 52:W83-W94.
7. Chen S, Zhou Y, Chen Y, Gu J. fastp: an ultra-fast all-in-one FASTQ preprocessor. Bioinformatics 2018; 34:i884-i890.
8. Anders S, Huber W. Differential expression analysis for sequence count data. Genome Biology 2010; 11:R106.
9. Gu Z, Eils R, Schlesner M. Complex heatmaps reveal patterns and correlations in multidimensional genomic data. Bioinformatics 2016; 32:2847–9.
10. Durinck S, Spellman PT, Birney E, Huber W. Mapping identifiers for the integration of genomic datasets with the R/Bioconductor package biomaRt. Nature Protocols 2009; 4:1184–91.
11. Xie Z, Bailey A, Kuleshov MV, Clarke DJB, Evangelista JE, Jenkins SL et al. Gene Set Knowledge Discovery with Enrichr. Curr Protoc 2021; 1:e90.
12. Zheng GXY, Terry JM, Belgrader P, Ryvkin P, Bent ZW, Wilson R et al. Massively parallel digital transcriptional profiling of single cells. Nat Commun 2017; 8:14049.
13. Melsted P, Booeshaghi AS, Liu L, Gao F, Lu L, Min KHJ et al. Modular, efficient and constant-memory single-cell RNA-seq preprocessing. Nat Biotechnol 2021; 39:813–8.
14. Wolf FA, Angerer P, Theis FJ. SCANPY: large-scale single-cell gene expression data analysis. Genome Biology 2018; 19:15.
15. Satija R, Farrell JA, Gennert D, Schier AF, Regev A. Spatial reconstruction of single-cell gene expression data. Nat Biotechnol 2015; 33:495–502.
16. Traag VA, Waltman L, van Eck NJ. From Louvain to Leiden: guaranteeing well-connected communities. Sci Rep 2019; 9:5233.
17. McInnes L, Healy J, Saul N, Großberger L. UMAP: Uniform Manifold Approximation and Projection. JOSS 2018; 3:861.

18. Badia-I-Mompel P, Vélez Santiago J, Braunger J, Geiss C, Dimitrov D, Müller-Dott S et al. decoupleR: ensemble of computational methods to infer biological activities from omics data. Bioinform Adv 2022; 2:vbac016.
19. La Manno G, Soldatov R, Zeisel A, Braun E, Hochgerner H, Petukhov V et al. RNA velocity of single cells. Nature 2018; 560:494–8.
20. Gayoso A, Weiler P, Lotfollahi M, Klein D, Hong J, Streets A et al. Deep generative modeling of transcriptional dynamics for RNA velocity analysis in single cells. Nat Methods 2024; 21:50–9.
21. Bergen V, Lange M, Peidli S, Wolf FA, Theis FJ. Generalizing RNA velocity to transient cell states through dynamical modeling. Nat Biotechnol 2020; 38:1408–14.
22. Weiler P, Lange M, Klein M, Pe'er D, Theis F. CellRank 2: unified fate mapping in multiview single-cell data. Nat Methods 2024; 21:1196–205.
23. Wolf FA, Hamey FK, Plass M, Solana J, Dahlin JS, Göttgens B et al. PAGA: graph abstraction reconciles clustering with trajectory inference through a topology preserving map of single cells. Genome Biology 2019; 20:59.
24. Aibar S, González-Blas CB, Moerman T, Huynh-Thu VA, Imrichova H, Hulselmans G et al. SCENIC: single-cell regulatory network inference and clustering. Nat Methods 2017; 14:1083–6.
25. Huynh-Thu VA, Irrthum A, Wehenkel L, Geurts P. Inferring regulatory networks from expression data using tree-based methods. PLoS ONE 2010; 5:e12776.
26. Moerman T, Aibar Santos S, Bravo González-Blas C, Simm J, Moreau Y, Aerts J et al. GRNBoost2 and Arboreto: efficient and scalable inference of gene regulatory networks. Bioinformatics 2019; 35:2159–61.
27. van de Sande B, Flerin C, Davie K, Waegeneer M de, Hulselmans G, Aibar S et al. A scalable SCENIC workflow for single-cell gene regulatory network analysis. Nature Protocols 2020; 15:2247–76.
28. Amirkhah R, Gilroy K, Malla SB, Lannagan TRM, Byrne RM, Fisher NC et al. MmCMS: mouse models' consensus molecular subtypes of colorectal cancer. Br J Cancer 2023; 128:1333–43.
29. Hoshida Y. Nearest template prediction: a single-sample-based flexible class prediction with confidence assessment. PLoS ONE 2010; 5:e15543.
30. Eide PW, Bruun J, Lothe RA, Sveen A. CMScaller: an R package for consensus molecular subtyping of colorectal cancer pre-clinical models. Sci Rep 2017; 7:16618.
31. Weise A, Bruser K, Elfert S, Wallmen B, Wittel Y, Wöhrle S et al. Alternative splicing of Tcf7l2 transcripts generates protein variants with differential promoter-binding and transcriptional activation properties at Wnt/beta-catenin targets. Nucleic Acids Res 2010; 38:1964–81.
32. Goldman MJ, Craft B, Hastie M, Repečka K, McDade F, Kamath A et al. Visualizing and interpreting cancer genomics data via the Xena platform. Nat Biotechnol 2020; 38:675–8.
33. Hunter JD. Matplotlib: A 2D Graphics Environment. Comput. Sci. Eng. 2007; 9:90–5.
34. Waskom M. seaborn: statistical data visualization. JOSS 2021; 6:3021.
35. RStudio Team. RStudio: Integrated Development for R.: Boston, MA; 2015. Available from: URL: http://www.rstudio.com/.

36. Wickham H. ggplot2: Elegant graphics for data analysis. Second edition. Cham: Springer international publishing; 2016. (Use R!). Available from: URL: https://ebookcentral.proquest.com/lib/kxp/detail.action?docID=4546676.
37. Soneoka Y, Cannon PM, Ramsdale EE, Griffiths JC, Romano G, Kingsman SM et al. A transient three-plasmid expression system for the production of high titer retroviral vectors. Nucleic Acids Res 1995; 23:628–33.
38. Zuber J, Rappaport AR, Luo W, Wang E, Chen C, Vaseva AV et al. An integrated approach to dissecting oncogene addiction implicates a Myb-coordinated self-renewal program as essential for leukemia maintenance. Genes Dev 2011; 25:1628–40.
39. Freihen V, Rönsch K, Mastroianni J, Frey P, Rose K, Boerries M et al. SNAIL1 employs β-Catenin-LEF1 complexes to control colorectal cancer cell invasion and proliferation. Int J Cancer 2020; 146:2229–42.
